# Instability of a liquid sheet with viscosity contrast in inertial microfluidics

**DOI:** 10.1140/epje/s10189-021-00147-1

**Published:** 2021-11-29

**Authors:** Kuntal Patel, Holger Stark

**Affiliations:** Institut für Theoretische Physik, Technische Universität Berlin, Hardenbergstr. 36, 10623 Berlin, Germany

## Abstract

**Abstract:**

Flows at moderate Reynolds numbers in inertial microfluidics enable high throughput and inertial focusing of particles and cells with relevance in biomedical applications. In the present work, we consider a viscosity-stratified three-layer flow in the inertial regime. We investigate the interfacial instability of a liquid sheet surrounded by a density-matched but more viscous fluid in a channel flow. We use linear stability analysis based on the Orr–Sommerfeld equation and direct numerical simulations with the lattice Boltzmann method (LBM) to perform an extensive parameter study. Our aim is to contribute to a controlled droplet production in inertial microfluidics. In the first part, on the linear stability analysis we show that the growth rate of the fastest growing mode $\xi ^{*}$ increases with the Reynolds number $\text {Re}$ and that its wavelength $\lambda ^{*}$ is always smaller than the channel width *w* for sufficiently small interfacial tension $\Gamma$. For thin sheets we find the scaling relation $\xi ^{*} \propto mt^{2.5}_{s}$, where *m* is viscosity ratio and $t_{s}$ the sheet thickness. In contrast, for thicker sheets $\xi ^{*}$ decreases with increasing $t_s$ or *m* due to the nearby channel walls. Examining the eigenvalue spectra, we identify Yih modes at the interface. In the second part on the LBM simulations, the thin liquid sheet develops two distinct dynamic states: waves traveling along the interface and breakup into droplets with bullet shape. For smaller flow rates and larger sheet thicknesses, we also observe ligament formation and the sheet eventually evolves irregularly. Our work gives some indication how droplet formation can be controlled with a suitable parameter set $\{\lambda ,t_{s},m,\Gamma ,\text {Re}\}$.

**Graphical Abstract:**

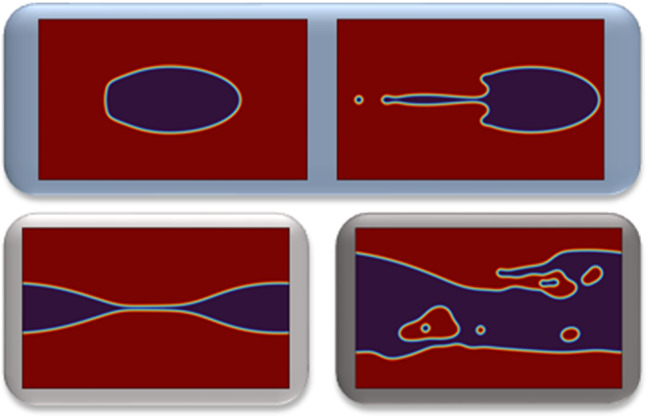

**Supplementary Information:**

The online version supplementary material available at 10.1140/epje/s10189-021-00147-1.

## Introduction

Miniaturized flow devices in the form of a lab-on-a-chip [1] are often employed for processing fluid flows on the micron scale [2]. Lab-on-a-chip microfluidic applications are used in cell biology [3], chemical synthesis [4], and for manipulating multi-component flows [5], to name but a few. Standard microfluidic devices operate in the Stokes flow regime, while only recently inertial microfluidic platforms have emerged [6]. Their flows at moderate Reynolds numbers enable high throughput and inertial focusing [7, 8] in order to develop manipulation techniques for biomedical applications. Motivated by this, a plethora of research has been carried out on inertial microfluidics in the last decade [9–13] including our own studies on the manipulation of soft capsules and solid particles using the inertial lift force [14–16].

**Fig. 1:**
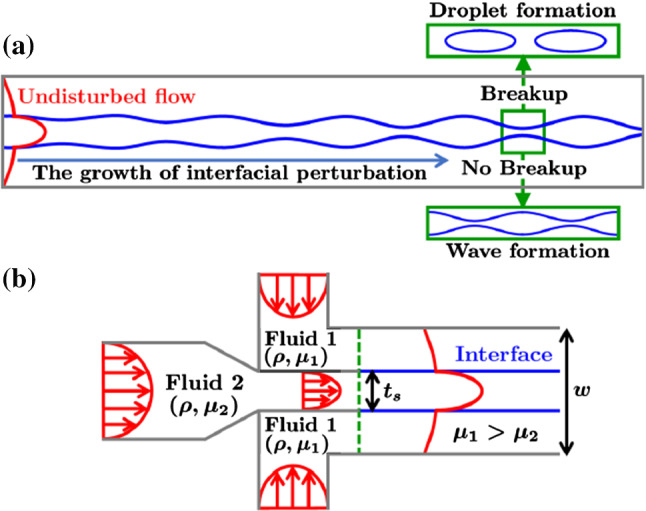
**a** Schematic of how an interfacial instability develops along a three-layer flow with viscosity contrast resulting in a steady interfacial wave or the formation of droplets. **b** Typical design of a channel inlet to generate a three-layer flow. The dashed green line indicates, where the channel walls separating fluid 1 and 2 ends and where the fluid–fluid interface begins

Recently, instabilities of single-phase flow in different geometries have also been investigated in the inertial regime with the aim to enhance fluid mixing [17, 18]. In this article, we use linear stability analysis and lattice Boltzmann simulations to investigate the viscosity-driven instability of a multi-component microfluidic flow at finite Reynolds numbers. We let a liquid sheet stream at the center of a microchannel surrounded by a flowing liquid of larger viscosity and same density and monitor its instability towards modulated interfaces and droplet breakup. Figure 1a shows how the instability develops along the flow direction in a sufficiently long channel. In contrast, in our theoretical investigation we will assume periodic boundary conditions. Such three-layer configurations with two interfaces are commonly encountered in two-phase microfluidic flows [19].

Yih [20] first showed that the fluid–fluid interface in two-layer Couette and Poiseuille flows with viscosity contrast is unstable irrespective of the value of the Reynolds number. Later studies concentrated on interface perturbations with small wavelengths [21, 22]. In general, instabilities in viscosity-stratified flows can occur either due to the direct presence of the fluid interface but also due to bounding walls. Boomkamp and Miesen presented an energy budget analysis for the unstable Yih or interface mode, which is triggered by the discontinuity of the shear rate at the interface [23]. Already single-phase flows become unstable at sufficiently large Reynolds numbers due to the presence of bounding walls which cause destabilizing Reynolds stresses. The resulting shear or Tollmien–Schlichting modes also exist in viscosity-stratified flows. Different energy contributions in the energy budget analysis of Boomkamp and Miesen [23] were quantified for two-layer channel flows by Valluri et al. [24] using linear stability analysis. Various nonlinear mechanisms governing the instability of viscosity-stratified flows were reported by Ó Náraigh et al. [25] using three-dimensional direct numerical simulations. Recently, Kalogirou et al. presented the interface dynamics of a thin viscous film adjacent to a wall in a two-layer channel flow with small viscosity contrast [26].

In addition to planar configurations, also core-annular flows in cylindrical channels have been investigated [27–31]. A recent linear stability analysis of core annular flows by Sahu [32] showed the existence of an unstable mode different from Yih and Tollmien–Schlichting modes, which Mohammadi and Smits [33] had also reported earlier in their linear stability analysis of two-layer Couette flows. Redapangu et al. [34] considered a two-phase flow in an inclined channel with the fluid–fluid interface of two immiscible fluids normal to the channel walls. In their numerical simulations they then studied how one fluid intrudes the other so that a very irregular three-layer flow arose. For more details on the instability of viscosity-stratified flows, we refer the reader to the comprehensive review article by Govindarajan and Sahu [35].

Viscosity-stratified flows naturally occur in microfluidics when droplets are generated. We review some relevant work. Kurdzinski et al. [36] working in the inertial regime reported different behavior of the central stream in a three-layer configuration of miscible fluids. With increasing Reynolds number they observed a disturbed, a broken, an oscillating, and a stable central stream. In their experiments at low to moderate Reynolds numbers, Hu and Cubaud [37] studied two-layer flows of miscible and immiscible fluids. They observed a linear relation between the wave frequency and the interface velocity. They could describe the dispersion relationship of the interfacial wave using capillary theory in the long-wave regime. Sengupta et al. [38] performed linear stability analysis of miscible fluids with viscosity contrast in rotationally actuated microfluid platforms in order to study fluid mixing. Already in 2007, Guillot et al. [39] investigated the formation of jets and droplets in core-annular microfluidic flows of immiscible fluids at low Reynolds numbers. Microfluidic experiments of Hu and Cubaud [40] addressed the formation of droplets via dripping and jetting in a quadratic microchannel with a less viscous center fluid. Moreover, at relatively high flow rates they only observed waves along the interface. Most recently, Dinh and Cubaud [41] also studied the effect of surface tension continuing work in Ref. [40] but with coaxial microchannels. A detailed review of various active and passive drop generation techniques in microfluidics can be found in Ref. [42]. In particular, external forces are used for active drop generation [43].

In the present work, we investigate the viscosity-driven instability of the fluid–fluid interface in a three-layer Poiseuille flow. We perform our work in the framework of inertial microfluidics also with the idea to contribute to controlled droplet generation by tuning fluid properties and geometry appropriately. We perform a thorough parameter study by varying sheet thickness, viscosity ratio, interfacial tension, and flow rate. In particular, we are also interested how the instability develops for different wavelengths of the interfacial perturbation as control parameter. In an experimental setting as sketched in Fig. 1b, an interfacial perturbation with a defined wavelength can be imposed by applying an electric field [44] or an oscillating pressure drop [45] across the inlet of the center fluid. Note, these mechanisms are required only for initial perturbation. The remaining process is solely governed by hydrodynamics. We also note that in this article, we study a two-dimensional flow configuration, which can be realized in microchannels with a large aspect ratio.

In the first part of our numerical study we perform a linear stability analysis based on the Orr–Sommerfeld equation and obtain dispersion relations of the unstable interface mode for various combinations of sheet thickness, viscosity ratio, interfacial tension, and Reynolds number. Furthermore, we discuss the structure of the eigenvalue spectra obtained by solving the Orr–Sommerfeld equation in the form of a generalized eigenvalue problem. In the second part, we present direct numerical simulations based on the lattice Boltzmann method (LBM). Starting from an interfacial perturbation of wavelength $\lambda$, for thin central sheets we observe two distinct dynamic states of the unstable fluid–fluid interface: traveling interfacial waves and generation of droplets via breakup of the fluid interface. Instead, for thicker sheets we observe interfacial waves but also ligament formation, where the sheet ultimately develops a very irregular shape instead of breaking up into droplets. Thus our study demonstrates the advantage of thin central sheets for controlled droplet generation. We also perform direct numerical simulations with multi-mode perturbations and show that they result in traveling waves with irregular shape.

The outline of the present article is as follows. In Sect. 2, we present the computational setup and the theory of the linear stability analysis along with the modeling of multi-component flows using the lattice Boltzmann method. We also benchmark our solvers for the linear stability analysis and the lattice Boltzmann simulations. Section 3 presents the numerical results of the linear stability analysis and the direct numerical simulations. We conclude with a summary and final remarks in Sect. 4.

## Problem setup and numerical methodology

### Computational domain

**Fig. 2:**
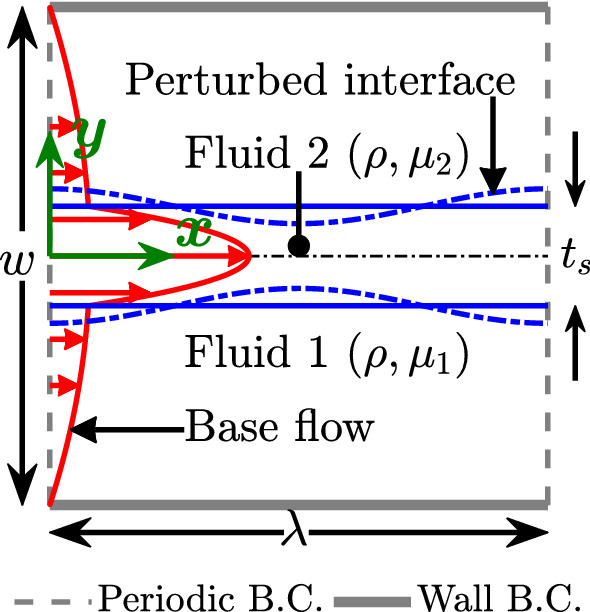
Computational setup for studying the instability of a liquid sheet. A microfluidic channel of width *w* and length $\lambda$ is considered, where $\lambda$ is also the wavelength of the interfacial perturbation. The liquid sheet of thickness $t_{s}$, density $\rho$, and viscosity $\mu _{2}$ is placed at the center of the microfluidic channel. The rest of the channel is filled with the fluid of the same density and viscosity $\mu _{1}$, where $\mu _{1}>\mu _{2}$. The surface tension of the fluid interface is $\sigma$

The microfluidic setup for the present problem is shown in Fig. 2. We consider a microfluidic channel with no-slip boundary condition at the bounding top and bottom walls of the channel and periodic boundary condition in the *x*-direction. The width and length of the channel are set to *w* and $\lambda$, respectively. $\lambda$ is also the wavelength of the interfacial perturbation to be studied in the following. To simplify the numerical implementation of the linear stability analysis, we will only consider perturbations symmetric about the channel center with an appropriate symmetry boundary condition at the channel center. However, direct numerical simulations are performed in the entire domain with no-slip boundary condition at the top and bottom channel walls. This allows us to capture a possible symmetry breaking of the liquid sheet driven by the instability.

We place the liquid sheet of thickness $t_{s}$, density $\rho$, and viscosity $\mu _{2}$ at the center of the microfluidic channel and fill the remaining part with another more viscous fluid of viscosity $\mu _{1}$ but matched by density. Solving the Navier–Stokes equation with constant magnitude of the pressure gradient $\gamma$ and interfacial boundary conditions

1 $$\begin{aligned} U_{\text {Fluid 1}}= & {} U_{\text {Fluid 2}} \nonumber \\ \mu _{1}\left. {\frac{\mathrm{d}U}{\mathrm{d}y}}\right| _{\text {Fluid 1}}= & {} \mu _{2}\left. {\frac{\mathrm{d}U}{\mathrm{d}y}}\right| _{\text {Fluid 2}}, \end{aligned}$$

so that flow velocity *U* and shear stress are continuous at the two interfaces, we arrive at the flow profile symmetric about the channel center at $y=0$:

2 $$\begin{aligned} U(y)= {\left\{ \begin{array}{ll} \displaystyle \frac{\gamma w^{2}}{8\mu _{1}} \bigg ( 1 - 4 \frac{y^{2}}{w^{2}} \bigg ) = \frac{\gamma w^{2}}{8\mu _{1}} U_1 &{} \text {Fluid 1} \\ \displaystyle \frac{\gamma w^{2}}{8\mu _{2}} U_2 &{} \text {Fluid 2}, \\ \end{array}\right. } \end{aligned}$$

with

3 $$\begin{aligned} U_2 = \frac{t_s^2}{w^{2}} + \frac{\mu _2}{\mu _1} \bigg (1 - \frac{t_s^2}{w^{2}}\bigg ) - 4\frac{y^2}{w^2} . \end{aligned}$$

Finally, to characterize the strength of the flow profile and the surface tension $\sigma$ of the interface, when introducing dimensionless quantities in our equations, we define the Reynolds number and the inverse capillary number as

4 $$\begin{aligned} \text {Re}_{c} = \frac{\rho U_{c}w}{\mu _{2}} \quad \mathrm {and} \quad \Gamma _{c} = \frac{\sigma }{\mu _{2}U_{c}}, \end{aligned}$$

where $U_{c}$ is a characteristic flow velocity. We choose $U_{c} = \gamma w^{2} / 8\mu _1$, which according to Eqs. (2) and (3) is the maximum flow velocity for $t_s \rightarrow 0$. We also take $\mu _2$ as the viscosity scale and for later use introduce the viscosity ratio $m = \mu _1/\mu _2$. From now on, we rescale all lengths by the channel width *w* and, in particular, use the dimensionless parameter $t_s / w \rightarrow t_s$ to quantify the sheet thickness.

In the following we consider two types of perturbations of the fluid–fluid interface. For the single-mode perturbation the interface is perturbed symmetrically about the channel center with an initial amplitude of $10^{-3}$. For the multi-mode perturbation we superimpose multiple cosine waves with random phase and amplitude $10^{-3}$.

### Computational linear stability analysis

To set up the linear stability analysis, we introduce a small perturbation of the base solution introduced in Eq. (2) and linearize the mass continuity equation and the Navier–Stokes equations in this perturbation. We name the non-dimensional perturbation for pressure *p* and we use *u*, *v* for the *x*, *y* components of the perturbations in the velocity flow field. Using the fact that the base solution satisfies the Navier–Stokes equations, we arrive at the linearized equations written in non-dimensional form:

5 $$\begin{aligned} \frac{\partial u_{n}}{\partial x}+\frac{\partial v_{n}}{\partial y}= & {} 0, \end{aligned}$$

6 $$\begin{aligned} \frac{\partial u_{n}}{\partial t}+ {U_n} \frac{\partial u_{n}}{\partial x}+v_{n}\frac{\partial {U_n}}{\partial y}= & {} -\frac{\partial p_{n}}{\partial x} + \frac{m_{n}}{\text {Re}_c}\nabla ^{2}u_{n}, \end{aligned}$$

7 $$\begin{aligned} \frac{\partial v_{n}}{\partial t}+ {U_n}\frac{\partial v_{n}}{\partial x}= & {} -\frac{\partial p_{n}}{\partial y} + \frac{m_{n}}{\text {Re}_c}\nabla ^{2}v_{n}, \end{aligned}$$

where $m_1 = m = \mu _1/\mu _2$ and $m_2 =1$ refer to fluids 1 and 2, respectively, and $U_n$ are the dimensionless flow profiles introduced in Eqs. (2) and (3).

We now formulate the Orr–Sommerfeld equation for both fluid phases, which is the basis for the linear stability analysis. For the velocity components in fluid 1 and 2 we introduce the stream functions $\psi _n$ such that the perturbations are written as

8 $$\begin{aligned} u_n = \frac{\partial \psi _n}{\partial y} \quad \mathrm {and} \quad -v_n = \frac{\partial \psi _n}{\partial x}. \end{aligned}$$

For stream function and pressure perturbation in both fluids, we make a plane wave ansatz along the channel axis and varying amplitude along the *y* direction:

9 $$\begin{aligned}{}[\psi _n,p_n]= & {} [\varphi _n(y),g_n(y)]e^{i k {(x-ct)}} , \end{aligned}$$

where *k* and *c* represent the real wave number and complex wave speed, respectively. The base flow is linearly unstable if growth rate $\xi =\text {Im}(k c)$ is positive. Upon substituting the ansatz functions in the linearized Eqs. (6) and (7) and eliminating the pressure gradient terms, we arrive at the well-known Orr–Sommerfeld equations for fluids 1 and 2:

10 $$\begin{aligned}&(a_{n}U_{n}-c)({\varphi }_n^{\prime \prime }-k^{2}\varphi _n)-a_{n} U^{\prime \prime }_{n}\varphi _n \nonumber \\&\quad =-\frac{i m_n}{k \text {Re}_c}(\varphi _n^{\prime \prime \prime \prime }-2k^{2}\varphi _n^{\prime }+ k^{4}\varphi _n) , \end{aligned}$$

where $$a_1=1$$ and $$a_2 = m = \mu _1 / \mu _2$$.

The linear stability analysis of the present problem requires the solution of Eq. (10) along with the following boundary conditions:

- No-slip boundary condition at the walls at $y=\pm 1/2$:
  11 $$\begin{aligned} \varphi _{1}=\varphi _{1}^{\prime }=0, \end{aligned}$$
- Symmetry boundary condition at $y=0$ for symmetric perturbations about the channel center:
  12 $$\begin{aligned} \varphi _{2}=\varphi _{2}^{\prime \prime }=0, \end{aligned}$$
- The continuity of *u* at both interfaces:
  13 $$\begin{aligned} \varphi _{1}^{\prime }+\frac{\varphi _{1} U^{\prime }_{1}}{c- U_{1}}=\varphi _{2}^{\prime }+\frac{\varphi _{2} m U^{\prime }_{2}}{c- m U_{2}}, \end{aligned}$$
- The continuity of *v* at both interfaces:
  14 $$\begin{aligned} \varphi _{1}=\varphi _{2}, \end{aligned}$$
- The continuity of shear stress at both interfaces:
  15 $$\begin{aligned} m\left[ \varphi _{1}^{\prime \prime }+k^{2}\varphi _{1}\right] =\varphi _{2}^{\prime \prime }+k^{2}\varphi _{2}, \end{aligned}$$
- The continuity of normal stress at both interfaces:
  16 $$\begin{aligned}&ik\text {Re}_c( U_{1}\varphi _{1}^{\prime } - m U_{2}\varphi _{2}^{\prime }) - m(\varphi _{1}^{\prime \prime \prime }-3k^{2}\varphi _{1}^{\prime }) \nonumber \\&\qquad + (\varphi _{2}^{\prime \prime \prime }-3k^{2}\varphi _{2}^{\prime }) -ik\text {Re}_c( U^{\prime }_{1}\varphi _{1}- m U^{\prime }_{2}\varphi _{2}) \nonumber \\&\quad =\frac{ik^{3}\Gamma _{c}}{ m U^{\prime }_{2}- U^{\prime }_{1}}\left( \varphi _{1}^{\prime }-\varphi _{2}^{\prime }\right) + ikc\text {Re}_c(\varphi _{1}^{\prime }-\varphi _{2}^{\prime }).\nonumber \\ \end{aligned}$$

In the present work, we employ the Chebyshev collocation method [46, 47] to obtain a numerical solution of the Orr–Sommerfeld equation. In the Chebyshev collocation method $\varphi _{n}(y)$ is approximated as

17 $$\begin{aligned} \varphi _{n}(y) = \sum ^{N_{n}}_{i=0}{a^{n}_{i}T^{n}_{i}(\eta _{n})}, \end{aligned}$$

where $T^{n}_{i}(\eta _{n})=\text {cos}( i~\text {cos}^{-1}(\eta _{n}))$ is the $i^{\text {th}}$ Chebyshev polynomial of the first kind. The application of the Chebyshev collocation method requires a linear transformation of the global *y*-coordinate (see Fig. 2) into local coordinates $\eta _{1}$, $\eta _{2}$ of fluids 1 and 2 such that $\eta _{1}$, $\eta _{2}~\in ~[-1,1]$:

18 $$\begin{aligned} \eta _{1}=\frac{-4|y|+ t_{s}+1}{t_{s}-1} \quad \text {and} \quad \eta _{2}=\frac{4|y|}{t_{s}}-1. \end{aligned}$$

Derivatives of $\varphi _{n}(y)$ needed in Eqs. (10)–(16) are obtained by differentiating Eq. (17) and using the linear transformation.

Once the number of collocation points $N_{1}$ and $N_{2}$ are fixed, the local coordinates of $N_{n}-3$ interior collocation points are

19 $$\begin{aligned} \eta _{n}=\text {cos}\left( \frac{\pi j}{N_{n}-2}\right) ,~\text {where}~j=[1,\ldots ,N_{n}-3].\nonumber \\ \end{aligned}$$

Now, we evaluate the Orr–Sommerfeld equation at the interior collocation points in the liquid sheet and the surrounding fluid using the trial solution of Eq. (17) and obtain $N_{1}+N_{2}-6$ equations with $N_{1}+N_{2}+2$ unknowns. The system of equations is closed by introducing the eight boundary conditions of Eqs. (11)–(16). Finally, the entire problem can be presented in the form of a generalized eigenvalue problem:

20 $$\begin{aligned}{}[A]\mathbf {\Phi }=-ikc[B]\mathbf {\Phi }, \end{aligned}$$

where [*A*] and [*B*] are complex square matrices of dimension $N_{1}+N_{2}+2$ and $\mathbf {\Phi }$ is a vector of the same dimension. In the present work, we use the eigenvalue solver provided by Matlab$^{\circledR }$ to obtain the solution of Eq. (20).

### Lattice Boltzmann method for multi-component flows

Direct numerical simulations of multi-component flows [48] require the solution of the mass and momentum conservation laws in each fluid component. Moreover, one also needs to trace the temporal evolution of the interface separating different fluid components. We start with the latter and then address lattice Boltzmann simulations of the fluid flow.

#### Interface dynamics in lattice Boltzmann simulations

Several techniques exist to access the time evolution of interfaces. On the one hand, they include tracking methods based on Lagrangian coordinates such as front tracking [49] and Lagrangian–Eulerian methods [50]. On the other hand, there also exist capturing methods based on Eulerian coordinates such as the volume-of-fluid [51], the diffuse [52] or sharp [53] interface level-set, and the phase-field [54] method. Interface capturing methods employ a scalar field to distinguish between two fluid components and have the inherent capability to realize the breakup of the fluid–fluid interface. In the present work, we implement the phase-field model based on the conservative Allen–Cahn equation (CACE):

21 $$\begin{aligned}&\frac{\partial \phi }{\partial t}+\nabla \cdot \left( \phi {\hat{{\mathbf {u}}}}\right) \nonumber \\&\quad =\nabla \cdot \left[ M\left( \nabla \phi -\frac{1-4{\left( \phi -0.5\right) }^{2}}{W}\frac{\nabla \phi }{|\nabla \phi |}\right) \right] ,\nonumber \\ \end{aligned}$$

where $\phi \in [0,1]$ is the phase-field variable and $\phi = 0$ and 1 characterize the two pure fluid components. Furthermore, $\hat{{\mathbf {u}}}$ is the fluid velocity field, *M* a mobility, and *W* quantifies the thickness of the fluid–fluid interface. The Allen–Cahn equation can also be derived from a generalized advection equation for interfaces [55], which was then reformulated as the above continuity equation [56].

In steady state, the phase-field equation (21) is solved by the hyperbolic tangent profile

22 $$\begin{aligned} \phi ^{\text {eq}}(\zeta )=\frac{1}{2}+\frac{1}{2}\text {tanh}\left( \frac{2\zeta }{W}\right) , \end{aligned}$$

where $\zeta$ is the coordinate normal to the fluid–fluid interface [55]. For more details on the implications, which the hyperbolic tangent profile has in descriptions of the interface different from the phase-field method, please refer to Ref. [57].

The Allen–Cahn equation can be discretized using techniques from traditional computational fluid dynamics (CFD) such as finite-volume and finite-element methods. However, for the present work the mesoscale approach based on the lattice-Boltzmann method (LBM) is more appropriate [58–60]. In the last two decades the lattice Boltzmann method has emerged as a computationally efficient alternative to traditional CFD techniques, mainly due to the capability of handling multi-physics problems involving soft-matter and fluid flow and the relative ease of parallel implementation [58, 59, 61].

We implement the phase-field lattice Boltzmann equation proposed by Fakhari et al. [62] on a uniform grid. The method uses the distribution function $h_{i}$ for the phase-field variable $\phi$, where the index *i* refers to a specific lattice-Boltzmann velocity vector ${\mathbf {c}}_{i}$. The distribution evolves through successive steps of collision,

23 $$\begin{aligned} {{\hat{h}}}_{i}({\mathbf {x}},t)={{h}}_{i}({\mathbf {x}},t)-\frac{h_{i}({\mathbf {x}},t)-h^{\text {eq}}_{i}({\mathbf {x}},t)}{\tau _{\phi }+0.5} , \end{aligned}$$

and advection,

24 $$\begin{aligned} h_{i}({\mathbf {x}},t+\Delta t)={\hat{h}}_{i}(\mathbf {x-{\mathbf {c}}}_{i},t) , \end{aligned}$$

where the equilibrium phase-field distribution function obeys

25 $$\begin{aligned} h^{\text{ eq }}_{i}= & {} {} \phi w_{i}\left[ 1+3{\left( \hat{{\mathbf {u}}} \cdot \mathbf {c}_{i}\right) }+\frac{9}{2}\left( \hat{{\mathbf {u}}} \cdot \mathbf {c}_{i}\right) ^{2}-\frac{3}{2}\left( \hat{{\mathbf {u}}} \cdot {\hat{\mathbf {u}}}\right) \right] \nonumber \\&+\,3w_{i}M\left[ \frac{1-4{(\phi - 0.5)}^{2}}{W}\right] \left[ {{\mathbf {c}}}_{i}\cdot \left( \frac{\nabla \phi }{|\nabla \phi |}\right) \right] .\nonumber \\ \end{aligned}$$

$\tau _{\phi }$ in Eq. (23) represents the relaxation time, which can be related to the mobility as $M=\tau _{\phi }/3$. Note that while deriving the Allen–Cahn equation (21) [55, 56], the curvature-driven motion is discarded using the counter-term approach proposed by Folch et al. [63]. Hence, *M* is a purely numerical parameter in our calculations. Note, the present LBM simulations are performed using the D2Q9 lattice with 9 discrete velocity vectors ${{\mathbf {c}}}_{i}=\{(0,0),\text {cyc}(\pm 1,0),(\pm 1,\pm 1)\}$ and corresponding weights $w_{i}=\{4/9,1/9,1/36\}$.

Now, we can calculate the phase-field variable $\phi$ since it is the zeroth moment of the distribution function $h_{i}$:

26 $$\begin{aligned} \phi = \sum ^{8}_{i=0}h_{i}. \end{aligned}$$

Finally, we compute the gradient of the phase-field variable $\nabla \phi$ in second-order accuracy using the isotropic central difference scheme [62, 64, 65]:

27 $$\begin{aligned} \nabla \phi = \frac{3}{2}\sum ^{8}_{i=1}w_{i}{\mathbf {c}}_{i}\left[ \phi ({\mathbf {x}}+{\mathbf {c}}_{i}, t)-\phi ({\mathbf {x}}-{\mathbf {c}}_{i}, t)\right] . \end{aligned}$$

#### Lattice Boltzmann model for fluid flow

The lattice Boltzmann equation governing the transport of the distribution function $f_{i}$ for the discrete velocity vectors ${\mathbf {c}}_i$ and in the presence of body forces ${\mathbf {F}}$ can be written as [66]

28 $$\begin{aligned} \frac{\partial f_{i}}{\partial t} + {\mathbf {c}}_{i}\cdot \nabla f_{i}=\Lambda (f^{\text {eq}}_{i}-f_{i}) + 3\Theta _{i}\left[ ({\mathbf {c}}_{i}-\hat{{\mathbf {u}}})\cdot {\mathbf {F}}\right] ,\nonumber \\ \end{aligned}$$

where $\Lambda$ is a collision operator to be introduced below and $f^{\text {eq}}_{i}$ is the discrete Maxwell distribution: $f^{\text {eq}}_{i} = \rho \Theta _{i}$ with

29 $$\begin{aligned} \Theta _{i} = w_{i}\left[ 1+3{\left( \hat{{\mathbf {u}}} \cdot {\mathbf {c}}_{i}\right) }+\frac{9}{2}\left( \hat{{\mathbf {u}}} \cdot {\mathbf {c}}_{i}\right) ^{2}-\frac{3}{2}\left( \hat{{\mathbf {u}}} \cdot {\hat{\mathbf {u}}}\right) \right] .\nonumber \\ \end{aligned}$$

In general, the density $\rho$ in $f^{\text {eq}}_{i}$ is calculated as an average over the two fluid densities $\rho _1$ and $\rho _2$ using the phase field, $\rho =\phi (\rho _{1}-\rho _{2})+\rho _{2}$, where $\rho _{1}>\rho _{2}$. However, in our case we consider density-matched fluids (see Fig. 2). Finally, the last term in Eq. (28) is a forcing scheme proposed by He et al. [67].

The body force ${\mathbf {F}}$ can be decomposed into three parts [66]:

30 $$\begin{aligned} {\mathbf {F}}=\nabla (\frac{\rho }{3}- {\hat{p}} )+{\mathbf {F}}_{s}+{\mathbf {F}}_{b}, \end{aligned}$$

where ${\hat{p}}$ is the pressure in lattice units and ${\mathbf {F}}_{b}$ is an external force acting on the fluid. The first term $\nabla (\frac{\rho }{3}- {\hat{p}})$ arises because close to the interface the LB fluid is no longer ideal. The force ${\mathbf {F}}_{s}$ is due to the surface tension of the fluid interface and in the present work following Ref. [68] is modeled as

31 $$\begin{aligned} {\mathbf {F}}_{s}=\mu _{\phi }\nabla \phi , \end{aligned}$$

with the chemical potential [69, 70]

32 $$\begin{aligned} \mu _{\phi }=\frac{48\sigma }{W}\left[ \phi (\phi -0.5)(\phi -1)\right] -\frac{3\sigma W}{2}\nabla ^{2}\phi . \end{aligned}$$

An alternative definition of the surface tension force uses ${\mathbf {F}}_{s}=-\phi \nabla \mu _{\phi }$ [71, 72]. However, Eq. (31) is the most suitable way for implementing ${\mathbf {F}}_{s}$ in a computer code since it avoids the necessity to calculate third-order derivatives of $\phi$. Following Fakhari et al. [65], we discretize $\nabla ^{2}\phi$ in Eq. (32) using

33 $$\begin{aligned} \nabla ^{2}\phi =6\sum ^{8}_{i=1}w_{i}\left[ \phi ({\mathbf {x}}+{\mathbf {c}}_{i}, t)-\phi ({\mathbf {x}}, t)\right] , \end{aligned}$$

which is accurate up to second-order.

To improve the numerical stability of our lattice Boltzmann scheme [62, 66, 73], we introduce a new distribution function

34 $$\begin{aligned} g_{i}=\frac{f_{i}}{3} + w_{i}\left( {\hat{p}}-\frac{\rho }{3}\right) . \end{aligned}$$

Substituting it in Eq. (28) and using Eq. (30) for the body force, we obtain the pressure-evolution lattice Boltzmann equation,

35 $$\begin{aligned} \frac{\partial g_{i}}{\partial t} + {\mathbf {c}}_{i}\cdot \nabla g_{i} = \Lambda (g^{\text {eq}}_{i}-g_{i}) + \Theta _{i} ({\mathbf {c}}_{i}-\hat{{\mathbf {u}}})\cdot {\mathbf {F}}_{g} \end{aligned}$$

which looks the same as Eq. (28) but with a modified body force ${\mathbf {F}}_g$:

36 $$\begin{aligned} {\mathbf {F}}_{g} = {\mathbf {F}}_{b} + \left[ \frac{(\Theta _{i}-w_{i})(\rho _{1}-\rho _{2})}{3 \Theta _i}+\mu _{\phi }\right] \nabla \phi , \end{aligned}$$

where in our case $\rho _1=\rho _2$ and the respective term in ${\mathbf {F}}_{g}$ vanishes.

In the present work, we employ the multiple-relaxa- tion-time (MRT) collision operator

37 $$\begin{aligned} \Lambda ={\mathbf {M}}^{-1}\hat{{\mathbf {S}}}{\mathbf {M}} , \end{aligned}$$

where $\hat{{\mathbf {S}}}$ is the diagonal relaxation matrix of the following form [62]:

38 $$\begin{aligned} \hat{{\mathbf {S}}}=\text {diag}\{1,1,1,1,1,1,1,1/\tau ,1/\tau \} . \end{aligned}$$

Following the Gram–Schmidt procedure [59], it is transformed using the transformation matrix for the D2Q9 lattice:

39 $$\begin{aligned} {\mathbf {M}}=\begin{pmatrix} 1 &{} 1 &{} 1 &{} 1 &{} 1 &{} 1 &{} 1 &{} 1 &{} 1\\ -4 &{} -1 &{} -1 &{} -1 &{} -1 &{} 2 &{} 2 &{} 2 &{} 2\\ 4 &{} -2 &{} -2 &{} -2 &{} -2 &{} 1 &{} 1 &{} 1 &{} 1\\ 0 &{} 1 &{} 0 &{} -1 &{} 0 &{} 1 &{} -1 &{} -1 &{} 1\\ 0 &{} -2 &{} 0 &{} 2 &{} 0 &{} 1 &{} -1 &{} -1 &{} 1\\ 0 &{} 0 &{} 1 &{} 0 &{} -1 &{} 1 &{} 1 &{} -1 &{} -1\\ 0 &{} 0 &{} -2 &{} 0 &{} 2 &{} 1 &{} 1 &{} -1 &{} -1\\ 0 &{} 1 &{} -1 &{} 1 &{} -1 &{} 0 &{} 0 &{} 0 &{} 0\\ 0 &{} 0 &{} 0 &{} 0 &{} 0 &{} 1 &{} -1 &{} 1 &{} -1 \end{pmatrix}. \end{aligned}$$

In the relaxation matrix $\hat{{\mathbf {S}}}$, $\tau =3\nu +0.5$ is the relaxation time and $\nu$ is the kinematic viscosity. To obtain the relaxation time within the fluid–fluid interface, we average the inverse time accordingly using the phase field $\phi$,

40 $$\begin{aligned} \frac{1}{\tau -0.5}=\phi \left( \frac{1}{\tau _{1}-0.5}-\frac{1}{\tau _{2}-0.5}\right) +\frac{1}{\tau _{2}-0.5} ,\nonumber \\ \end{aligned}$$

where $\tau _{1}$ and $\tau _{2}$ are the respective relaxation times of the two fluid phases.

As usual, one solves the pressure-evolution lattice Boltzmann equation (36) in two steps [62, 74]:

1. *Collision step*
  41 $$\begin{aligned} \hat{g_{i}}({\mathbf {x}},t)= & {} {\bar{g}}_{i}({\mathbf {x}},t)+ \Lambda [{\bar{g}}^{\text {eq}}_{i}({\mathbf {x}},t)-{\bar{g}}_{i}({\mathbf {x}},t)] \nonumber \\&+\, \Theta _{i} ({\mathbf {c}}_{i}-\hat{{\mathbf {u}}})\cdot {\mathbf {F}}_{g} \end{aligned}$$
  where we introduced the pressure distribution function
  42 $$\begin{aligned} {\bar{g}}_{i}({\mathbf {x}},t)= & {} g_{i}({\mathbf {x}},t)-\frac{1}{2}\hat{{\mathbf {S}}}[{g}^{\text {eq}}_{i}({\mathbf {x}},t)-{g}_{i}({\mathbf {x}},t)]\nonumber \\&-\frac{1}{2} \Theta _{i} ({\mathbf {c}}_{i}-\hat{{\mathbf {u}}})\cdot {\mathbf {F}}_{g} \end{aligned}$$
  and the expression for ${\bar{g}}^{\text {eq}}_{i}({\mathbf {x}},t)$ is obtained by replacing $g_i$ and ${{\bar{g}}}_i$ by their respective equilibrium distributions $g^{\text {eq}}_{i}$ and ${\bar{g}}^{\text {eq}}_{i}$ in the previous formula. Note, the transformation of the pressure distribution function $g_{i}({\mathbf {x}},t)$ into ${\bar{g}}_{i}({\mathbf {x}},t)$ using Eq. (42) in the collision and the following advection steps is performed to make the scheme explicit in time. [62, 65].
2. *Advection step*
  43 $$\begin{aligned} {\bar{g}}_{i}({\mathbf {x}},t+\Delta t)={\hat{g}}_{i}(\mathbf {x-{\mathbf {c}}}_{i},t). \end{aligned}$$

The directional derivative of the phase-field variable, ${\mathbf {c}}_{i}\cdot \nabla \phi$, which appears in the collision step when calculating $({\mathbf {c}}_{i}-\hat{{\mathbf {u}}})\cdot {\mathbf {F}}_{g}$, is discretized using the mixed finite difference approximation [62, 75],

44 $$\begin{aligned} {\mathbf {c}}_{i}\cdot \nabla \phi ({\mathbf {x}},t)= & {} \frac{1}{4}\left[ -\phi ({\mathbf {x}}-{\mathbf {c}}_{i},t)-3\phi ({\mathbf {x}},t)\right. \nonumber \\&+ \left. 5\phi ({\mathbf {x}}+{\mathbf {c}}_{i},t)-\phi ({\mathbf {x}}+2{\mathbf {c}}_{i},t)\right] .\nonumber \\ \end{aligned}$$

The isotropic central difference scheme with the same accuracy is used for computing the modified equilibrium distribution function ${\bar{g}}^{\text {eq}}_{i}({\mathbf {x}},t)$:

45 $$\begin{aligned} {\mathbf {c}}_{i}\cdot \nabla \phi ({\mathbf {x}},t) = \frac{1}{2}\left[ \phi ({\mathbf {x}}+{\mathbf {c}}_{i},t)-\phi ({\mathbf {x}}-{\mathbf {c}}_{i},t)\right] . \end{aligned}$$

Finally, the macroscopic quantities such as momentum density $\rho \hat{{\mathbf {u}}}$ and pressure ${\hat{p}}$ are calculated from the pressure distribution function ${{\bar{g}}}_i$:

46 $$\begin{aligned} \rho \hat{{\mathbf {u}}}= & {} 3\left[ \sum ^{8}_{i=0}{\bar{g}}_{i}{\mathbf {c}}_{i}+\frac{1}{2}\left( \mu _{\phi }\nabla \phi +{\mathbf {F}}_{b}\right) \right] ,\nonumber \\ {\hat{p}}= & {} \sum ^{8}_{i=0}{\bar{g}}_{i}+\frac{1}{6}\left( \rho _{1}-\rho _{2}\right) (\hat{{\mathbf {u}}}\cdot \nabla \phi ). \end{aligned}$$

In our lattice Boltzmann simulations, we set the interface width *W* to 4 lattice units and use a bounce-back rule to implement a no-slip boundary condition at the channel walls [59, 62].

### Simulation parameters

The instability of the liquid sheet is governed by its thickness, the wavelength of the interfacial perturbation, viscosity ratio, Reynolds number, and inverse capillary number. In this subsection, we discuss the values of these parameters, which we take for the linear stability analysis and direct numerical simulations.

Before proceeding, we introduce the mean flow velocity $U_b$ in addition to the previously defined velocity scale $U_{c}=\gamma {w}^{2}/8\mu _{1}$. The velocity scale $U_{c}$ is convenient in theory for making the governing equations non-dimensional. However, to connect the present numerical simulations to microfluidic experiments, the mean flow velocity is more appropriate since it directly determines the volumetric flow rate $Q=U_{b}{w}$. Thus, for the following discussions we use the new Reynolds number $\text {Re}$ and inverse capillary number $\Gamma$ based on the velocity scale $U_{b}$. They are readily linked to $\mathrm {Re}_c$ and $\Gamma _c$ introduced in Eq. (4) using the flow profiles from Eqs. (2) and (3) to calculate $U_b$ from the flow rate *Q*:

47 $$\begin{aligned} \text {Re}= & {} \frac{\rho U_{b} w}{\mu _{2}} = \text {Re}_{c} \, \frac{2}{3}\left[ t^{3}_{s} (m -1)+1 \right] , \end{aligned}$$

48 $$\begin{aligned} \Gamma= & {} \frac{\sigma }{\mu _2 U_{b}} = \Gamma _{c} \, \frac{3}{2}\left[ t^{3}_{s} (m -1)+1 \right] ^{-1} . \end{aligned}$$

We will perform an extensive parameter study to obtain a complete overview on the behavior of unstable interfacial perturbations using realistic parameter values. In the linear stability analysis we vary the Reynolds number $\text {Re}$ from 10 to 500 in steps of 5 and the reduced wavelength $\lambda$ of the interfacial perturbation from 0.01 to 5 in steps of $1.25\times 10^{-3}$, quasi continuously. This will enable us to present the color coded growth rate of an unstable perturbation in the $\text {Re}$–$\lambda$ plane for two values of the non-dimensional sheet thickness, $t_{s}=0.1$ and 0.13, two viscosity ratios, $m=30$ and 100 and four values of the inverse capillary number, $\Gamma = \{10^{-2},10^{-1},1,10\}$. We will also investigate larger sheet thicknesses $t_{s}$ up to 0.5 at $\text {Re}=100$ and $\Gamma =0.01$. In direct numerical simulations on the time evolution of interfacial single-mode perturbations, we study all the combinations arising from $\lambda =\{0.25,0.5,0.75,1\}$ for $\text {Re} = 500$, with the same parameter values of $\Gamma$, $t_{s}$, and *m*, as mentioned before. The specific choices of the wavelength $\lambda$ and Reynolds number $\text {Re}$ is based on the outcome of the linear stability analysis and will become apparent in the next section. Exemplary simulations are also carried out at Re=100. Moreover, we use the same values of $t_{s}$, *m*, and $\text {Re}$ for direct numerical simulations of multi-mode perturbations using $\Gamma =10^{-2}$.

In typical inertial microfluidic applications, where particles are manipulated using the inertial lift force, the channel Reynolds number varies in the range $1 - 100$. [76]. We are well in this range, when we define a proper Reynolds number. For example, when we concentrate on the thin liquid sheet, we need to replace in Eq. (47) the channel width *w* by the sheet thickness $t_s$. Or to describe the outer fluid using the higher viscosity $\mu _1$ is more appropriate. An alternative would be to replace the viscosity $\mu _2$ by averaging the viscosity over the whole channel cross section with the two fluid components, which then gives the Reynolds number $\text {Re}[t_{s}(m-1)+1]m^{-1}$. In the present setup for all the possible combinations of $\mathrm {Re}$, $t_s$, and *m* this multi-component Reynolds number is below 100.

In experiments one of the possible ways to realize the setup shown in Fig. 2 is by appropriately merging the outlets of three different microchannels containing the top (fluid 1), middle (fluid 2), and bottom (fluid 1) layers, as sketched in Fig. 1b. In this case the size of the microchannel outlet delivering fluid 2 will determine the sheet thickness $t_{s}$. The small thickness-to-width ratio $t_s$ chosen in our work is achievable in experiments since microchannels with widths ranging from 16 to $500\ \mu \text {m}$ were employed in experiments [77, 78]. Furthermore, the viscosity ratio *m* can roughly range from 1 to 1000, which justifies our choice of $m=30$ and 100. For example, pure water ($\mu = 1 \, \text {cP}$) and different silicone oils ($\mu =9.3$, 97, and $971 \, \text {cP}$) cover a range of the viscosity ratio from ca. 10 to 1000 [79]. This makes our choice of high viscosity ratios realistic. Finally, in microfluidic experiments at $\text {Re} \ll 1$ the inverse capillary number $\Gamma$ can assume values of order 1 or larger, *e.g.*, by choosing ethanol and silicone oil with $m=45$ and $\sigma =0.65$ mN/m [40]. In an inertial microfluidic setup, the bulk velocity $U_{b}$ is larger and inverse capillary numbers, $\Gamma \propto U^{-1}_{b}$, below one are feasible. Hence, also the range of $\Gamma$ in the present work is justified.

### Validation and grid convergence

**Fig. 3:**
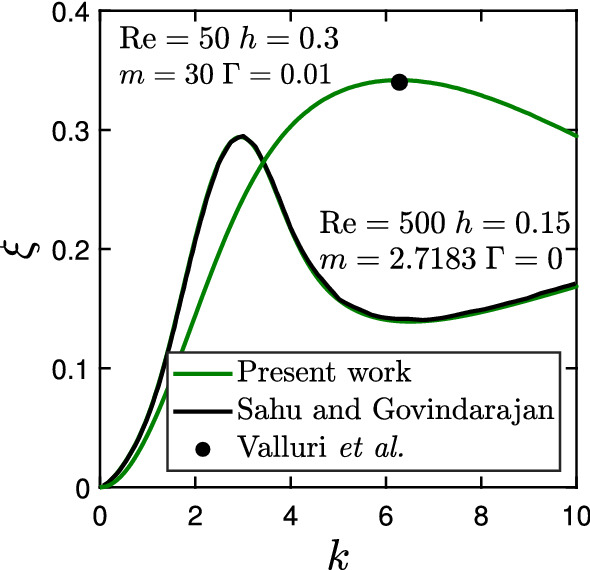
Validation of the dispersion curves, growth rate $\xi$ versus wave number *k*, with published results on the instability in two-layer flows

To validate our solver for the linear stability analysis, we consider a two-layer flow with just one interface in a channel of height 1, where the bottom layer has viscosity $\mu _{1}$ and thickness *h*. The viscosity of the less viscous top layer is $\mu _{2}$. Note, this problem can be transformed into our original problem (see Fig. 2) by replacing the no-slip boundary condition with the symmetry boundary condition at the top domain boundary. We address two cases with $\{\text {Re},h,m,\Gamma \}=\{500,0.15,{e=2.718},0\}$ and $\{50,0.3,30,0.01\}$. In both cases, we use 51 collocation points in each fluid layer. The dispersion curves, growth rate $\xi$ versus wave number *k*, obtained from our solver for both cases are plotted in Fig. 3 along with the dispersion curve reported by Sahu and Govindarajan [80] for the first case and the fastest growing mode from Valluri et al. [81] for the second case. It is evident from Fig. 3 that our results are in very good agreement with published works. In particular, our dispersion curve and the one from Ref. [80] lie exactly on top of each other.

We now focus our attention on the validation and grid convergence of the present lattice-Boltzmann solver for the setup shown in Fig. 2. We select two sets of parameters: $\{\lambda ,\text {Re},t_{s},m,\Gamma \} = \{0.5,500,0.13,100,0.01\}$ and $\{0.5,500,0.1,30,0.01\}$. In each case we compare the time evolution of the amplitude of the unstable mode obtained from lattice Boltzmann simulations with the result of the linear stability analysis using 51 collocation points in each fluid component. For lattice Boltzmann simulations we employ four grid resolutions: $N=128, 256, 512$, and 1024, where *N* is the number of grid points across the channel width.

**Fig. 4:**
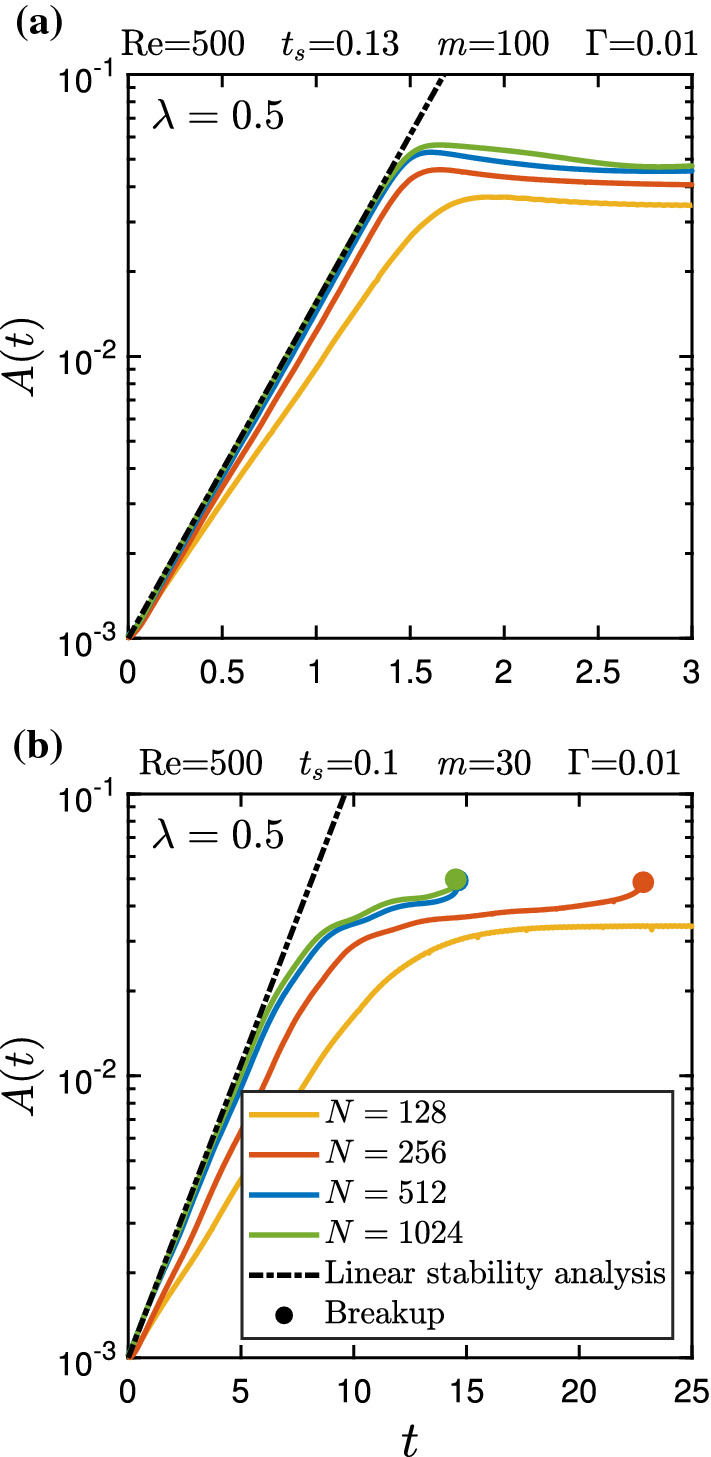
Time evolution of the amplitude of an unstable interface mode determined with the linear stability analysis and lattice Boltzmann simulations. *N* indicates the number of grid points across the channel width in lattice Boltzmann simulations

As shown in Fig. 4, the linear stability analysis and lattice Boltzmann simulations with $N=512$ and 1024 produce nearly identical results for the early growth of the interface disturbance. In the first case [see Fig. 4a], lattice Boltzmann simulations suggest that the perturbation grows initially and then reaches a plateau. In contrast, in the second case [see Fig. 4b] we observe the breakup of the liquid sheet. Finally, we conclude that the grid resolution of $N=512$ is good enough to capture the interfacial instability in the present problem using lattice Boltzmann simulations.

## Results and discussion

We begin with the results obtained from the linear stability analysis for the perturbed liquid sheet. Based on our findings, we then present results from lattice Boltzmann simulations for specific cases with single and multi-mode perturbations.

### Linear stability analysis

#### Parameter study of the interfacial mode

**Fig. 5:**
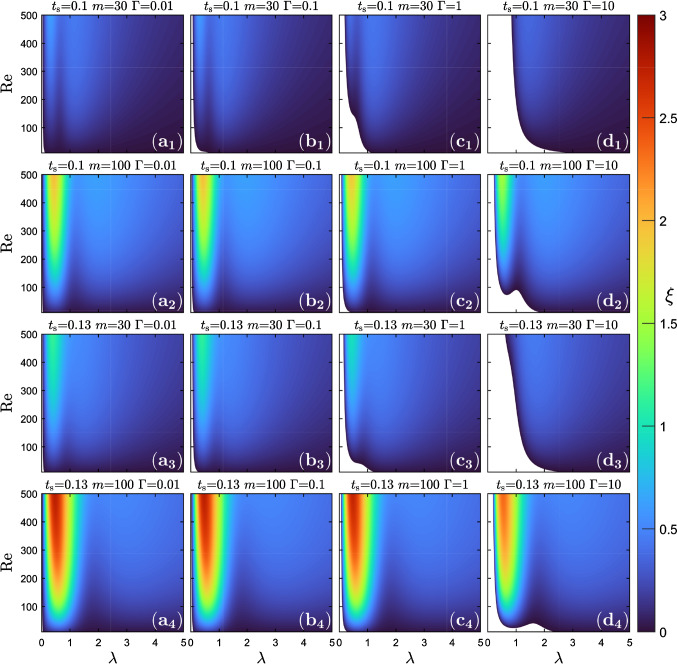
Color-coded growth rate $\xi$ in the $\text {Re}$–$\lambda$ plane for different values of the sheet thickness $t_{s}$, viscosity ratio *m*, and inverse capillary number $\Gamma$. Regions of stable modes with negative $\xi$ are marked in white

We first look at how our material parameters influence the instability of the liquid sheet when symmetric perturbations with wavelength $\lambda$ are applied at both interfaces. For this we performed an extensive parameter study and plot in Fig. 5 the color-coded growth rate $\xi$ in the $\text {Re}$–$\lambda$ plane for specific parameter combinations of the sheet thickness $t_{s}$, viscosity ratio *m*, and inverse capillary number $\Gamma$. Stable perturbations are indicated by white regions. We find that for all $\text {Re}$–$\lambda$ combinations, perturbations are unstable for flows with relatively weak interfacial tension [see Fig. 5($\text {a}_1$)–($\text {a}_4$)], while stable modes at small $\lambda$ appear in the columns 2-4, where $\Gamma$ is larger. Interestingly, in all cases except when larger white regions with stable modes exist, the wavelength associated with the fastest growing mode is always below the channel width, which is one in our reduced units. Moreover, the growth rate of the fastest growing mode always increases with the Reynolds number. The growth rate strongly depends on the wavelength for values smaller than the channel width. For wavelengths larger than the channel width, it reaches a plateau for constant Re.

**Fig. 6:**
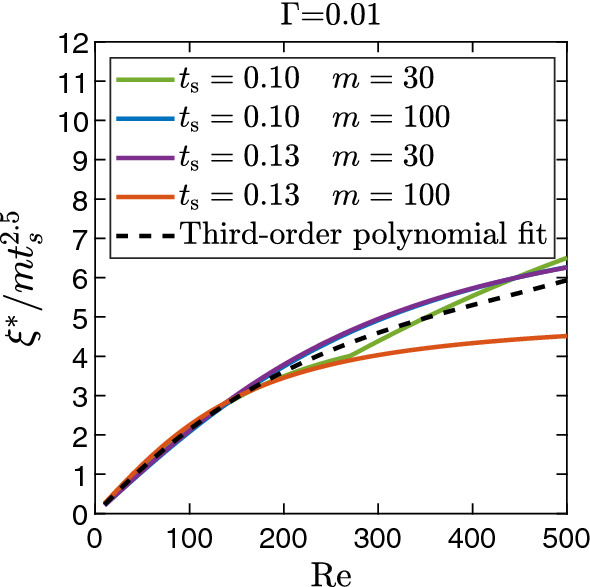
Master curve of the growth rate of the fastest growing mode $\xi ^{*}$ in units of $m t_s^{2.5}$ plotted versus $\text {Re}$ for weak interfacial tension $\Gamma = 0.01$ and several combinations of *m* and $t_s$. Note, the master curve fits well until ca. $\mathrm {Re} = 200$

Increasing the viscosity ratio *m* or the sheet thickness $t_s$, makes the fluid–fluid interface clearly more unstable, which can be qualitatively described as $\xi \propto m^{a}t^{b}_{s}$ ($a,b>0$). This shows that the present interfacial instability is strongly governed by the viscosity contrast. Most notably, increasing the sheet thickness from 0.1 to 0.13 seems to be a small variation, but it has a significant effect on the growth rate, as rows 3 and 4 in Fig. 5 show. The observed dependence is made more quantitative upon rescaling the growth rate of the fastest growing mode, $\xi ^{*}\rightarrow \xi ^{*}/mt^{2.5}_{s}$. Values of exponents *a* and *b* in the scaling law are obtained based on the best fit. The rescaled values plotted versus $\text {Re}$ nicely fall on a master curve for the same $\Gamma =0.01$ and $\text {Re}$ up to ca. 200 (see Fig. 6). The master curve is represented by a cubic polynomial fit and shown as a dashed line. While the curve for $t_s=0.13$ and $m=100$ strongly deviates from the master curve beyond $\text {Re}\approx 200$, the curves for the other parameters still roughly follow it. In particular, the rescaling with $t_s^{2.5}$ demonstrates the sensitivity of the most unstable mode on the sheet thickness.

**Fig. 7:**
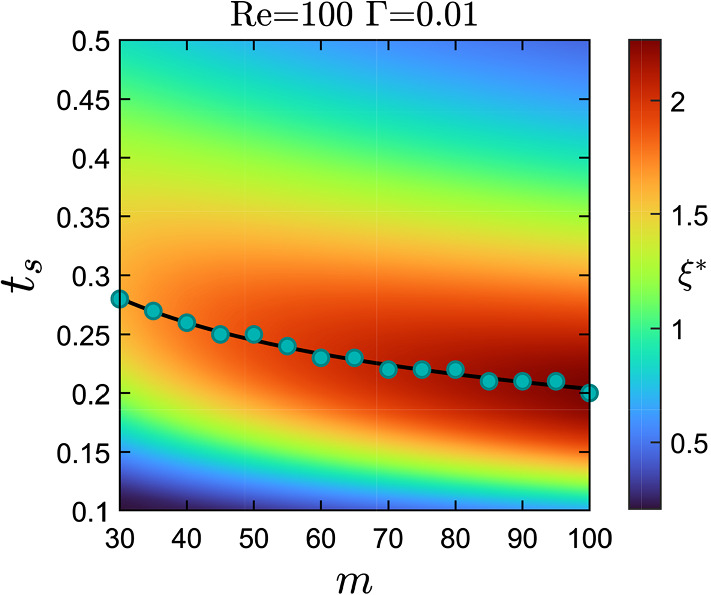
Variation of the growth rate of the fastest growing mode $\xi ^{*}$ in the *m*–$t_{s}$ parameter space at Reynolds number $\text {Re}=100$ and inverse capillary number $\Gamma =0.01$. Filled circles represent the critical thickness $t^{*}_{s}$ at which the growth rate $\xi ^{*}$ is maximal for a fixed viscosity ratio *m*. The solid black line is a power-law fit for the critical thickness: $t^{*}_{s}=0.7m^{-0.2675}$

We now focus our attention on investigating the instability over an extensive range of sheet thickness and viscosity ratio keeping Reynolds and inverses capillary numbers fixed. In order to study sheet thicknesses up to values of 0.5 relevant in microfluidic devices, we choose $\text {Re} = 100$ which keeps the relevant multi-component Reynolds number (see discussion in Sect. 2.4) below 100. In Fig. 7, we plot the color-coded growth rate of the fastest growing mode $\xi ^{*}$ in the *m*–$t_{s}$ plane. Interestingly, in contrast to what we observed so far that with increasing $t_s$ the instability of the interface becomes stronger, we now identify a critical sheet thickness $t^{*}_{s}$ beyond which the growth rate decreases. We speculate that this is due to interactions with the channel walls. The critical sheet thickness for different viscosity ratios is marked using filled circles in Fig. 7 and described by the fit curve $t^{*}_{s}=0.7m^{-0.2675}$ (solid black line). It quantifies the slow decrease of $t_s$ with increasing *m*.

**Fig. 8:**
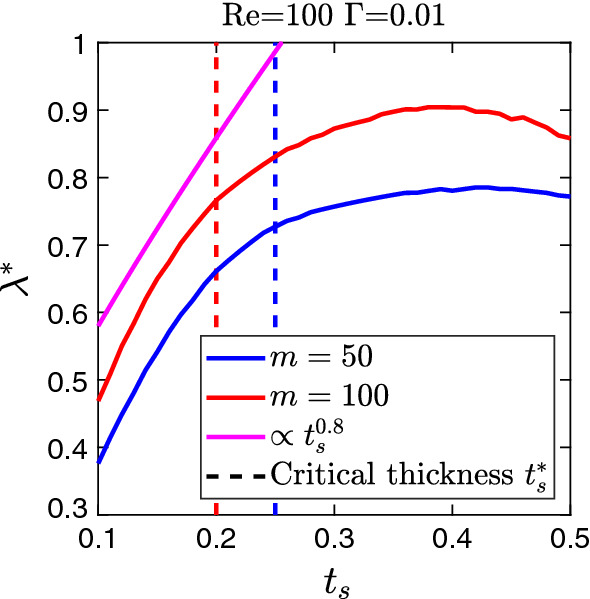
Wavelength of the fastest growing mode $\lambda ^{*}$ plotted versus the sheet thickness $t_{s}$ for viscosity ratios $m=50$ and 100 at Reynolds number $\text {Re}=100$ and inverse capillary number $\Gamma =0.01$

**Fig. 9:**
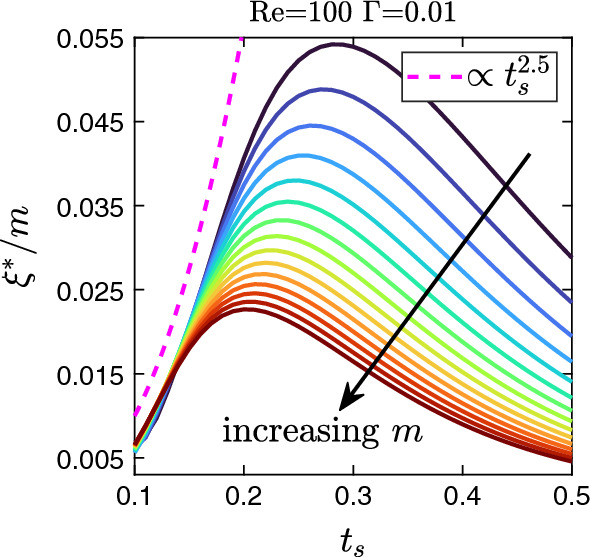
Rescaled growth rate of the fastest growing mode $\xi ^{*} / m$ plotted versus $t_{s}$ for different *m*. Dashed line: Scaling law $\xi ^{*} / m \propto t_s^{2.5}$

To explore the large range of sheet thicknesses further, we plot in Fig. 8 the wavelength of the fastest growing mode $\lambda ^{*}$ versus $t_s$ for viscosity ratios $m=50$ and 100. While for small $t_s$ below the critical thickness, the wavelength increases noticeably and roughly linearly, its variation above $t^{*}_{s}$ is much weaker. Similar trends are observed for the other viscosity ratios. Secondly, in Fig. 6 we showed that the growth rate $\xi ^{*}$ of the fastest growing mode scales with $m t^{2.5}_{s}$ up to $\text {Re} \approx 200$. In Fig. 9, we plot $\xi ^{*} / m$ versus $t_s$ for the full range from 0.1 to 0.5 for several *m* values. On the left of the maximum of $\xi ^{*} / m$, i.e., for small $t_s$, we confirm the scaling, while beyond the maximum it is no longer valid. In Fig. 9, we see that the scaling law starts to deviate as the sheet thickness approaches the critical value. For a fixed viscosity ratio, the value of the critical sheet thickness decreases with the increase in the Reynolds number (not shown here), which results in the early deviation of the scaling law at higher values of $\text {Re}$. This can be the possible cause for the departure of $t_{s}=0.13$ $m=100$ curve beyond $\text {Re=200}$ in Fig. 6.

#### Exemplary eigenvalue spectra

**Fig. 10:**
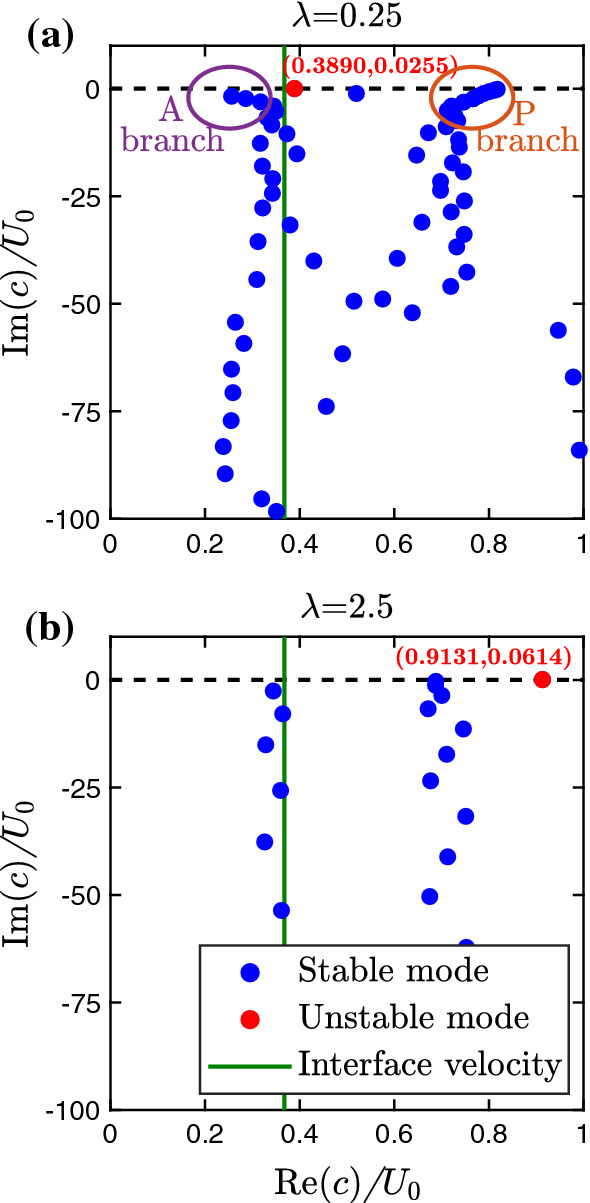
Eigenvalue spectra representing the complex wave speed *c* of flow perturbations for **a** $\lambda =0.25$ and **b** $\lambda =2.5$. The imaginary part $\text {Im}(c)= \xi /k$ is proportional to the growth rate, while $\text {Re}(c)$ is the wave speed, both given in units of centerline velocity $U_{0}$. The remaining parameters are $\text {Re}=500$, $t_{s}=0.13$, $m=100$, and $\Gamma =0.01$

So far we have concentrated on the unstable interface mode. For sufficiently small reduced surface tension $\Gamma$ it always occurs for a nonzero viscosity contrast, i.e., for $m \ne 1$ [20, 23]. Now, we present the full eigenvalue spectrum of modes, which we obtain for a given wavelength $\lambda$, when solving the Orr–Sommerfeld equations (10) in the form of the generalized eigenvalue problem of Eq. (20). For the parameters $\text {Re}=500$, $t_{s}=0.13$, $m=100$, and $\Gamma =0.01$ (bottom left of Fig. 5), we choose two wavelengths: $\lambda = 0.25$, which is close to the fastest growing mode, and $\lambda = 2.5$, which is less unstable. Figure 10 represents the complex wave speed of the perturbation modes introduced in Eq. (9), where $\text {Im}(c) = \xi / k$ with $k= 2\pi / \lambda$ is proportional to the growth rate and $\text {Re}(c)$ is the wave velocity.

In both cases, we observe only one unstable mode [red dot with positive $\text {Im}(c)$], which clearly belongs to the interface becoming unstable. All the other modes are stable [blue dots with negative $\text {Im}(c)$]. For $\lambda =0.25$ and small $|\text {Im}(c)|$, we identify A and P branches, which are reminiscent of the eigenvalue spectrum observed in the single-phase channel flow of a Newtonian fluid [47]. They form the typical upper left and upper right part of a Y-shaped structure in the eigenvalue spectrum called Tollmien–Schlichting modes and center modes, respectively. The modes travel with the respective speeds $\text {Re}(c) / U_{0}\rightarrow 0$ and $\text {Re}(c) / U_{0}\rightarrow 1$. As expected, for stable Poiseuille flow they are also stable.

The unstable interface mode is called Yih mode, named after the scientist who first described these modes quantitatively also in a Poiseuille flow with viscosity contrast [20, 23]. For $\lambda = 0.25$, the wave speed is close to the flow velocity of the interface, thus in the co-moving frame of the interface, the perturbation wave is static and only the amplitude grows. This changes, when we look at the wavelength $\lambda = 2.5$, larger than the channel width. First of all, in the stable modes we do not observe A and P branches. Instead, we recognize two vertical branches: one with wave speed very close to the interface velocity and another branch with a larger wave speed. Interestingly, the unstable interface mode has a wave speed close to the centerline velocity $U_0$. Hence, in a co-moving frame of the fluid–fluid interface, the wave is traveling with growing amplitude.

#### Energy-budget analysis

The unstable interface mode reveals itself also when looking at the energy budget of the disturbance flow [23, 24, 82]. Here, one considers the kinetic energy of the disturbance flow, which in our case amounts to $E(t)=\int \limits _{V} (u^{2}_{n}+v^{2}_{n})/2\,\mathrm{d}V$, where $V= \lambda \times w$ is the total volume. The base flow solution of the Navier–Stokes equations is stable if the disturbance kinetic energy $E(t) > 0$ converges to zero with time, meaning the base flow is restored. To decide on this, one calculates the time derivative $\mathrm{d}E/\mathrm{d}t$ from the Navier–Stokes equations as outlined in “Appendix A.” For a flow geometry with interfaces such as the three-layer flow, one obtains

49 $$\begin{aligned} \frac{\mathrm{d}E(t)}{\mathrm{d}t} = q_{\text {dis}}+q_{\text {Rey}}+q_{\text {nor}}+q_{\text {tan}} \end{aligned}$$

with four energy rates on the RHS associated with different mechanisms. The first two terms already exist in a single-phase flow. The rate $q_{\text {dis}} < 0$ denotes the typical energy dissipation through viscous friction, while $q_{\text {Rey}}$ results from the convective derivative in the Navier–Stokes equations. It couples Reynolds shear stresses to the base flow and when positive signals the presence of an unstable Tollmien–Schlichting mode. The third and the fourth terms occur when applying the continuity of normal and tangential shear stresses at the fluid interface, respectively. In the energy-budget analysis, the destabilizing disturbance mode can be assigned to one of the four energy rates driving the instability.

**Fig. 11:**
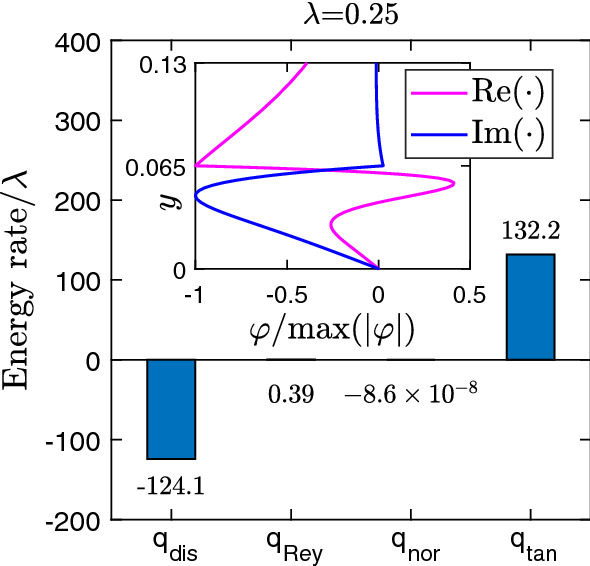
Values of the four energy rates (per channel length $\lambda$) from the energy-budget equation (A1) calculated for an unstable interface mode, which was determined in the linear stability analysis. Inset: Real and imaginary parts of the eigenfunction in the vicinity of the fluid–fluid interface at $y=0.065$. The remaining parameters are $\lambda = 0.25$, $\text {Re}=500$, $t_{s}=0.13$, $m=100$, and $\Gamma =0.01$

To illustrate the energy budget analysis, we apply it to the unstable mode identified in Fig. 10a. The stream function $\varphi$ is illustrated in the inset of Fig. 11. Since the *x* component of the velocity derives from the derivative of the stream function, $u_n \propto d\varphi _n / dy$, one realizes that the disturbance flow is concentrated at the interface. When calculating the different energy rates, we skipped the exponential time factor. Clearly, dissipation due to viscous friction ($q_{\text {dis}}$) is overcome by energy $q_{\text {tan}}$ fed into the system by the tangential stresses at the interface due to the viscosity contrast. Thus the interface mode is unstable. Negligible contributions arise from $q_{\text {Rey}} >0$ and $q_{\text {nor}} <0$. From a mathematical view, the destabilizing energy rate $q_{\text {tan}}$ is connected to the discontinuity in $u_{n} \propto d\varphi _n / dy$ (see “Appendix A”), which has its origin in the viscosity contrast.

#### Fluid–fluid interface close to the channel wall

**Fig. 12:**
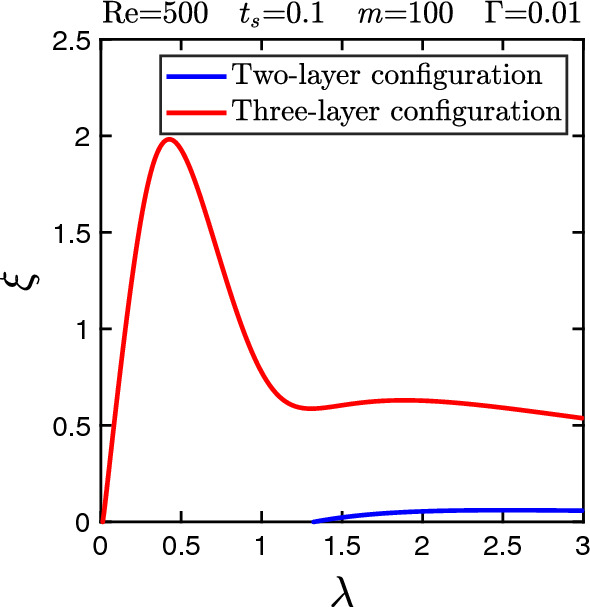
Comparison of the dispersion curves, growth rate $\xi$ versus wavelength $\lambda$, for two- and three-layer configurations. Other parameters are indicated in the plot

As a side remark, we consider the case where the thin liquid sheet is put in contact with one of the channel walls. Thus the current three-layer system transforms into a two-layer configuration with only one interface. We can also treat this geometry with our formalism by changing the relevant boundary condition. Figure 12 compares the growth rates as a function of $\lambda$ for the exemplary case of $\text {Re}=500$, $t_{s}=0.1$, $m=100$, and $\Gamma =0.01$. While the three-layer configuration shows the behavior as discussed before, we observe that the presence of the wall close to the interface makes the interface mode stable for wavelengths smaller than the channel width, but even at larger $\lambda$ the growth rate is considerably smaller. Interested readers may refer to Ref. [21] for further discussion on the neutral stability of interface modes in two-layer configurations.

**Fig. 13:**
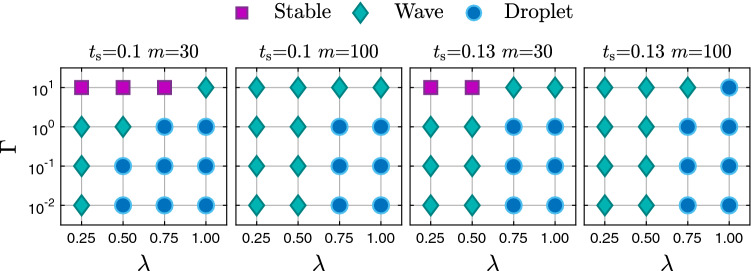
The liquid sheet is either stable against an imposed perturbation of wavelength $\lambda$, develops traveling waves along the two interfaces, or breaks up into droplets depending on inverse capillary number $\Gamma$, sheet thickness $t_{s}$, and viscosity ratio *m*. The Reynolds number is $\text {Re}=500$

### Direct numerical simulations

In this subsection, we present results obtained from direct numerical simulations (DNS) using the lattice Boltzmann method. We carry out DNS for two different values of the Reynolds number: $\text {Re}=100$ and 500. We begin with results from DNS of flows with $\text {Re}=500$ since, compared to $\text {Re} = 100$, it requires less computational time to obtain conclusive results for the small sheet thicknesses $t_{s}=0.1$ and 0.13. In a second step, based on the results for $\text {Re}=500$, we simulate flows at $\text {Re}=100$ for specific values of the perturbation wavelength and interfacial tension.

#### Flows with $\mathrm {Re}=500$

First, we discuss the case of single-mode perturbations and then proceed to multi-mode perturbations. The results of the linear stability analysis in Fig. 5 indicated strong variations of the growth rate $\xi$ for $\lambda \le 1$. In the following, we thus consider the wavelengths $\lambda =\{0.25,0.5,0.75,1\}$ and use the same combinations of $t_{s}$, *m*, and $\Gamma$ as in Fig. 5.

In Fig. 13, we summarize the dynamic states into which the perturbed interface evolves in the $\lambda$–$\Gamma$ plane. Only for the smaller viscosity ratio and the highest reduced surface tension $\Gamma$, the interface is stable in agreement with the findings in Fig. 5. Unstable perturbations evolve into traveling waves in the range of smaller wavelengths, which extends to larger values if $\Gamma$ is increased. In contrast, at larger wavelengths the interface breaks up and droplets form. We now proceed to a detailed discussion of the two dynamic states of propagating interface waves and droplet formation.

**Fig. 14:**
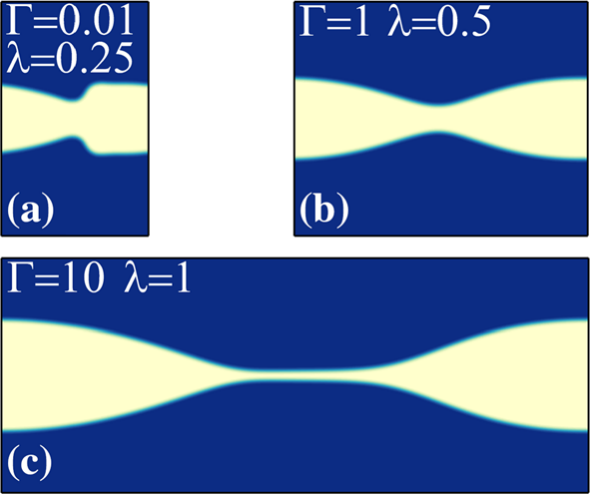
Steady-state wave patterns obtained from lattice Boltzmann simulations starting with an interfacial disturbance of wavelength $\lambda$ at different inverse capillary numbers $\Gamma$. The remaining parameters are $\text {Re}=500$, $t_{s}=0.1$, and $m=30$

**Fig. 15:**
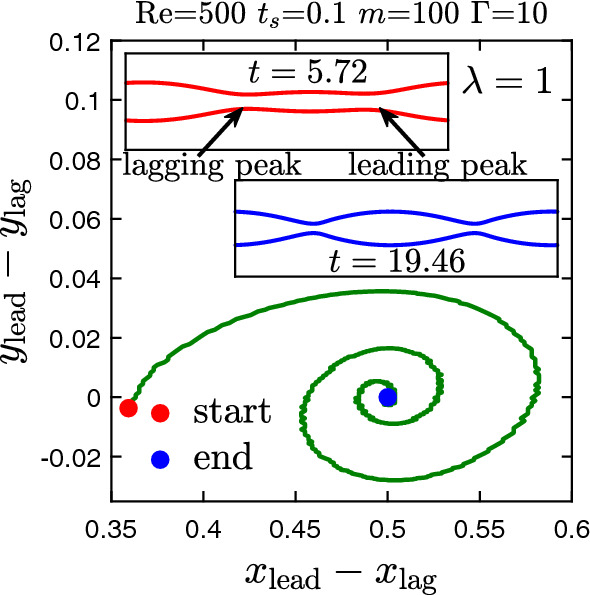
An interfacial perturbation of wavelength $\lambda =1$ develops a leading and lagging peak. The differences of their position coordinates along the stream-wise and the cross-stream direction are plotted in the *x*–*y* plane. Insets: Snapshots of the fluid–fluid interface at the start (top inset) and end (bottom inset) of the spiral

**Fig. 16:**
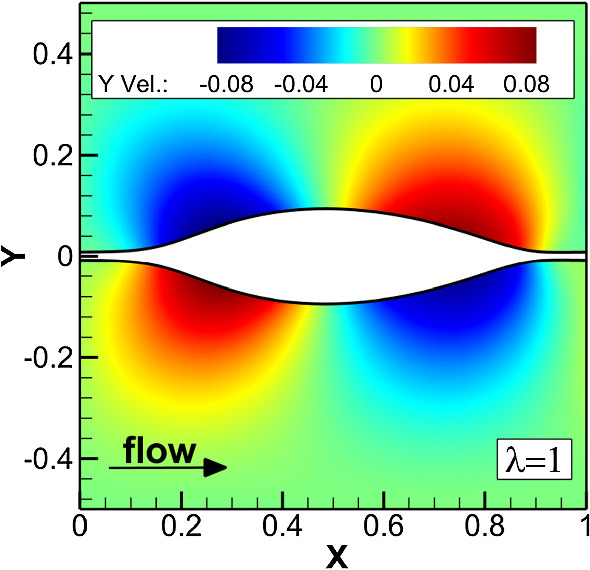
Color-coded representation of the *y* component of the disturbance velocity field in the fluid surrounding the liquid sheet at $\text {Re}=500$, $t_{s}=0.1$, $m=30$, and $\Gamma =10$

Figure 14 shows examples of steady-state wave patterns, which we observe for different $\lambda$ and $\Gamma$. For flows with weak interfacial tension ($\Gamma =0.01$) and small wavelength a sawtooth-shaped fluid interface develops. For $\Gamma =1$ and $\lambda =0.5$, we obtain an interface shaped like an hourglass, reminiscent of experimental results reported by d’Olce *et al*. [83] for core annular flows of two miscible fluids with a viscosity contrast. At our maximal value of the interfacial tension ($\Gamma =10$) and perturbation wavelength equal to the channel width ($\lambda =1$), we see two liquid blobs connected by a thin liquid thread [see Fig. 14c and video V1]. For all these cases the interface patterns preserve the wavelength of the initial perturbation. However, we observe an exception for parameters $t_{s}=0.1$, $m=100$, and $\Gamma =10$. For this particular setting, the initial perturbation ultimately develops into a secondary disturbance with half the initial wavelength $\lambda = 1$ (see video V2). At intermediate times we observe two weak peaks as shown in the top inset of Fig. 15. They oscillate relative to each other in the stream-wise and cross-stream directions. Upon plotting their relative coordinates in both directions, we obtain an inward spiral, as shown in the main plot. In steady state (blue dot in Fig. 15), both peaks attain the same lateral position and a distance of 0.5 along the flow. Thus, the initial perturbation has developed into a an interfacial wave with half the initial wavelength (see the bottom inset in Fig. 15). Finally, in Fig. 16 we show the disturbance velocity field in the *y*-direction for the wave pattern in Fig. 14c. Since the blob moves to the right, it has to shuffle fluid from the front to the back. The cross-stream motion of the viscous fluid in Fig. 16 can potentially be utilized to enhance heat transfer in liquid-liquid microfluidic flows [84].

**Fig. 17:**
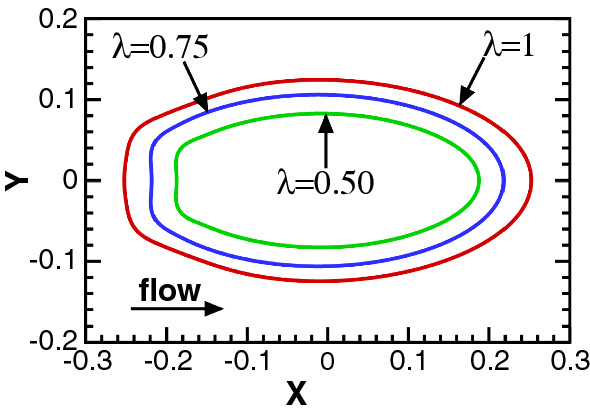
Final droplet shape after the breakup of the liquid sheet resulting from different perturbation wavelengths $\lambda$ at $\text {Re}=500$, $t_{s}=0.1$, $m=30$, and $\Gamma =0.01$

**Fig. 18:**
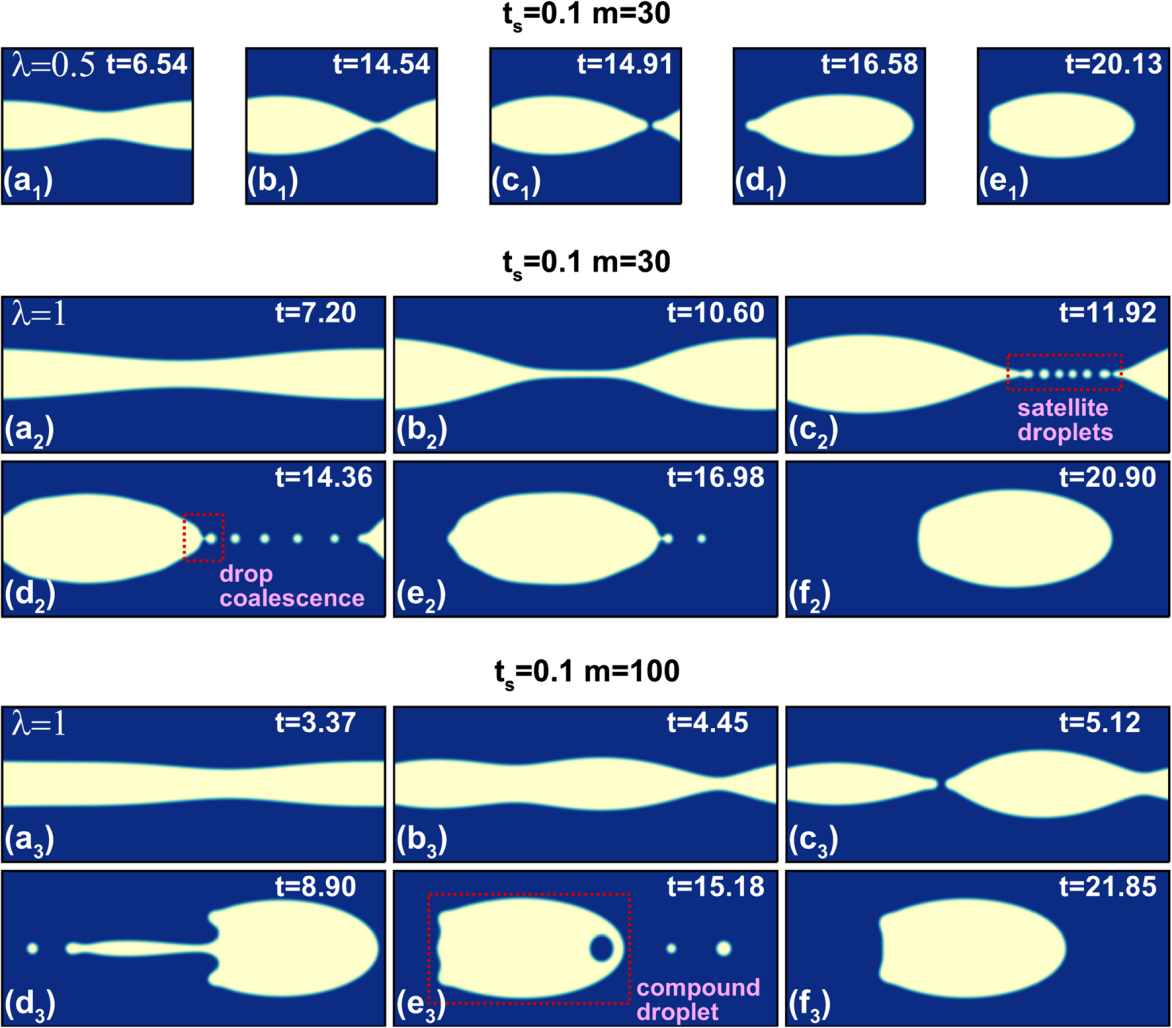
Influence of the perturbation wavelength $\lambda$ and viscosity ratio *m* on the time evolution of the liquid sheet and the breakup dynamics towards a bullet-shaped droplet at $\text {Re}=500$ and $\Gamma =0.01$

**Fig. 19:**
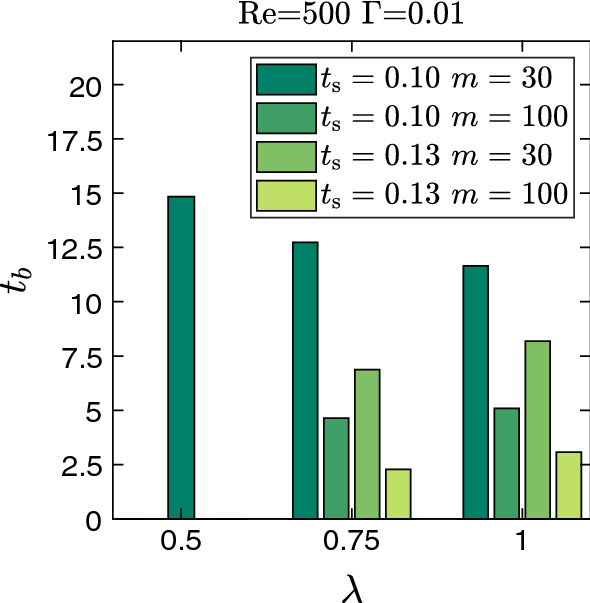
Interface breakup time $t_{b}$ of the liquid sheet towards droplet formation for different combinations of the perturbation wavelength $\lambda$, sheet thickness $t_{s}$, and viscosity ratio *m* at $\text {Re} = 500$ with weak interfacial tension

In the third dynamic state, the unstable interface breaks up and develops into bullet-shaped droplets of nearly the same aspect ratio around 2. We show this in Fig. 17 for specific parameters but observe similar behavior for other parameter values. However, the transient dynamics towards the final droplet can differ, as we illustrate in Fig. 18 where we show different stages of droplet generation for three representative cases. For $\lambda =0.5$ the breakup of the interface occurs due to the merger of the approaching wave crests. In contrast, for $\lambda =1$ and $m=30$, a thin liquid thread of less viscous fluid breaks into multiple satellite droplets, which then coalesce with the primary droplet (see video V3). At the higher viscosity ratio of $m=100$, we initially see an additional modulation with half the prescribed wavelength similar to what we described in Fig. 15. Instead of observing satellite droplets, we now obtain a droplet with a slender tail [see Fig. 18($\text {d}_{3}$)]. The tail eventually breaks and merges with the droplet behind it. During this process, the entrapment of a viscous droplet in the less viscous primary droplet occurs, as shown in Fig. 18($\text {e}_{3}$). Eventually, the entrapped droplet escapes from the tip of the primary droplet. Interestingly, in all three cases the bullet-shaped form of the droplets always develops at approximately the same time $t\approx 20$ (see Fig. 18) irrespective of their timing of sheet breakup. In Fig. 19, we summarize the breakup times $t_{b}$ of the liquid sheet for different parameter sets with relatively weak interfacial tension ($\Gamma =0.01$). At constant $\lambda$ we observe that an increase in either sheet thickness $t_{s}$ or viscosity ratio *m* leads to an earlier breakup, which coincides with the increase in the growth rate of the interfacial mode. We do not see any substantial change of this behavior at higher values of $\Gamma$. The viscosity-driven breakup of the liquid sheet in the present work can be utilized for a controlled droplet production in inertial microfluidics. At constant $\text {Re}$, droplet size and breakup time can be adjusted by selecting an appropriate parameter set $\{\lambda , t_{s}, m, \Gamma \}$.

**Fig. 20:**
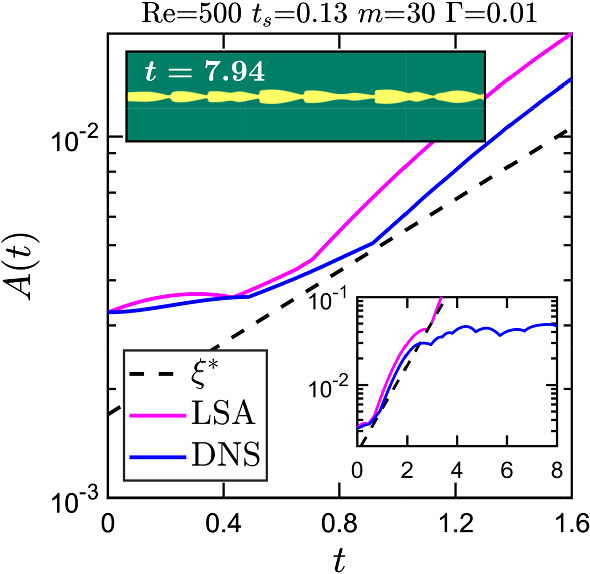
Time variation of the maximum interface displacement *A*(*t*) determined from direct numerical simulations (DNS) and the dispersion relations of the linear stability analysis (LSA) when the small multi-mode perturbation of Eq. (50) is imposed at $t=0$. The dashed black line $\xi ^{*}$ corresponds to the fastest growing mode. Bottom inset: Time evolution of *A*(*t*) at later time instances; top inset: snapshot of the fluid–fluid interface in steady-state

In the end we briefly discuss a generic case where we disturb the liquid sheet with a multi-mode perturbation. We use the same values of viscosity ratio *m* and sheet thickness $t_{s}$ from earlier cases of single-mode perturbation and set the inverse capillary number to $\Gamma =0.01$. We choose a channel with length $\lambda = 3$ and width $w=1$ and disturb the two interfaces symmetrically with a superposition of 15 planar waves:

50 $$\begin{aligned} \sum ^{15}_{n=1}5\times 10^{-4}\text {cos}\left( \frac{2\pi nx}{3}+ \theta _{n}\right) , \end{aligned}$$

where $\theta _{n}$ is a randomly generated phase. For a representative case the time evolution of the maximum displacement *A*(*t*) of one interface determined in the lattice-Boltzmann simulations is plotted in Fig. 20 along with the superposition of the 15 time-evolving modes from linear stability analysis. The dashed line indicates the growth rate of the fastest growing mode.

The LSA and DNS curves in Fig. 20 indicate three different stages of time evolution. In the beginning, both curves roughly lie on top of each other. The instability grows more slowly in comparison to the fastest growing mode (the dashed black line in Fig. 20). But then it speeds up since small wavelengths become effective and enhance the growth rate; from Fig. 5 we know that waves of small wavelengths grow faster than those with long wavelengths. In particular, in the LSA curve one recognizes that between $t\approx 0.5$ and 0.7 the fastest growing mode dominates before all the other modes further speed up the growth. The time evolution enters the second stage at $t\approx 0.7$, where both curves start to depart from each other obviously due to nonlinearities, which only the DNS curve takes into account. Interestingly, after $t\approx 1$ both curves grow with the same rate. In the third and final stage starting at $t\approx 2$ the DNS curve reaches a plateau as indicated in the bottom inset of Fig. 20, while the LSA curve grows further. The snapshot of the fluid–fluid interface from this stage shows a sawtooth-like interface profile as observed earlier in single-mode simulations but now the sequence is irregular (see video V4). We observe similar dynamics for other combinations of the sheet thickness $t_{s}$ and viscosity ratio *m*.

#### Flows with $\mathrm {Re}=100$

We further explore here the possibility to generate droplets, now at a smaller Reynolds number $\text {Re}=100$. We concentrate on a sheet thickness set to the critical value $t^{*}_{s}$, where the growth rate is largest, and consider our two viscosity ratios $m=30$ and 100. Furthermore, we choose the perturbation wavelength $\lambda =1$ and $\Gamma =0.01$.

**Fig. 21:**
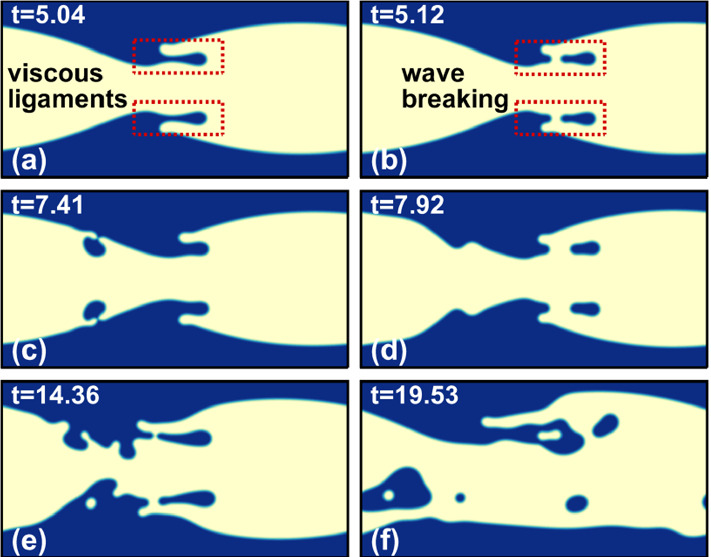
Formation of ligaments and enclosure of smaller droplets in an unstable liquid sheet of critical thickness $t^{*}_{s}=0.28$ with perturbation wavelength $\lambda =1$. At later time instances the mirror symmetry of the sheet breaks. The other parameters are viscosity ratio $m=30$, $\text {Re}=100$, and $\Gamma =0.01$

For $m=100$ and $t^{*}_{s}=0.2$, only traveling interfacial waves form and the fluid interface does not break. However, in the second case, for $m=30$ and $t^{*}_{s}=0.28$, the traveling wave develops ligaments close to the wave crest, as shown in Fig. 21a. Eventually, the ligaments connect back to the liquid sheet and thereby small droplets of the more viscous fluid are enclosed in the sheet [see Fig. 21b]. The formation and reconnection of ligaments continues while the small droplets get advected downstream and eventually merge with the interface, as shown in Fig. 21c (see video V5). This leads to a small local disturbance at the thinner part of the sheet [Fig. 21d]. Eventually, the mirror symmetry of the liquid sheet breaks and the fluid–fluid interface exhibits an irregular topology [Fig. 21e, f]. A similar phenomenon of ligament formation and irregular evolution has recently been observed in microfluidic experiments of Hu and Cubaud [40].

Interestingly, for thinner sheets with $t_{s}=0.1$ and $\{\lambda ,m\}=\{1,30\}$ we do observe the formation of a droplet although the instability is less pronounced compared to the discussed example. This indicates that a strong growth rate does not guarantee droplet formation. Instead, it is important that the sheet thickness is small enough to realize the breakup of the sheet such that droplets are able to form.

## Concluding remarks

Lab-on-a-chip devices based on inertial microfluidics operate in the regime of moderate Reynolds numbers where fluid flow is still regular. While conventional inertial microfluidic applications manipulate soft capsules and solid particles using the inertial lift force (see, for example, Ref. [16]), in this article we focused on multi-component flows in the inertial regime. Concretely, we studied the motion of a liquid sheet at the center of a microchannel surrounded by a flowing liquid of larger viscosity and monitored its instability towards traveling interfacial waves and droplet breakup. Such a configuration is ubiquitous in microfluidic applications involving multi-component flows.

In the first part, we presented dispersion relations for the interfacial mode of the three-layer configuration. The computational linear stability analysis was based on the Orr–Sommerfeld equation. We carried out an extensive parameter study to quantify the effect of sheet thickness $t_{s}$, viscosity ratio *m*, interfacial tension $\Gamma$, and Reynolds number $\text {Re}$ on the growth rate. In particular, we observed that the growth rate of the fastest growing mode $\xi ^{*}$ increases with the Reynolds number $\text {Re}$ and that its wavelength $\lambda ^{*}$ is always smaller than the channel width *w* for sufficiently small inverse capillary number $\Gamma$. Furthermore, we could quantify the dependence of $\xi ^{*}$ on *m* and $t_{s}$ by the scaling law $\xi ^{*} \propto mt^{2.5}_{s}$ in the case of thin sheets, moderate Reynolds numbers, and at weak interfacial tension. In contrast, for thick sheets $\xi ^{*}$ decreases with increasing sheet thickness $t_{s}$ or viscosity ratio *m* due to the presence of the channel walls. Upon examining eigenvalue spectra obtained by solving the generalized eigenvalue problem of Eq. (20), we concluded that Yih modes drive the present interfacial instability [20].

In the second part, we narrowed down the range of material parameters based on the linear stability analysis and presented numerical solutions of the full Navier–Stokes equations using lattice Boltzmann (LBM) simulations. At $\text {Re} = 500$ the thin liquid sheet was stable only for the smaller viscosity ratio and the highest reduced surface tension $\Gamma$. Unstable interfaces either evolve into traveling waves or for wavelengths $\lambda \ge 0.5w$ they break up and ultimately form droplets when interfacial tension is sufficiently small. The droplets eventually assume a bullet-shaped form. We found that for different perturbation wavelengths $\lambda$ and viscosity ratios *m* the temporal evolution towards the droplet differed in the observed interface dynamics and the way the sheet ultimately broke up. However, the droplet formation always ended roughly at the same time. In the introduction, we briefly explained how the wavelength of the interfacial perturbation can be controlled in an experiment.

In practical applications droplet size and breakup time can be tuned by selecting a suitable parameter set $\{\lambda ,t_{s},m,\Gamma \}$ at constant $\text {Re}$. In slower flows at $\text {Re}=100$ and for thicker liquid sheets, we observed the formation of viscous ligaments at the wave crest, which eventually break up into small droplets and ultimately the shape of the sheet becomes irregular. Thus, we conclude that controlled droplet formation is better achieved with thin sheets. Finally, in direct numerical simulations of multi-mode perturbations we demonstrated how the interface develops a non-regular shape.

In the present work, we investigated the viscosity-driven instability of the fluid–fluid interface in the context of inertial microfluidics and demonstrated that it can be exploited for controlled droplet production. Such droplets are utilized in food and pharmaceutical industries [85]. Moreover, microfluidic droplets are also employed to encapsulate biological cells [86] and as chemical reactors in lab-on-a-chip devices [87].

### Supplementary Information

Below is the link to the electronic supplementary material.

Supplementary material 1 (mp4 129 KB)

Supplementary material 2 (mp4 140 KB)

Supplementary material 3 (mp4 236 KB)

Supplementary material 4 (mp4 137 KB)

Supplementary material 5 (mp4 502 KB)

## Appendix A: Energy-budget analysis

We consider a volume *V* filled with multiple fluids separated by interfaces. We index each fluid component with *n* and its rescaled viscosity is $m_n$ as in the main text. We start with the Navier–Stokes equations for the full flow field as the sum of base and disturbance flow and multiply it with the disturbance velocity field $u_i^n$. Using Gauss theorem, the incompressibility condition, the no-slip boundary condition at the surface of *V*, and in our case also periodic boundary conditions, one arrives at the budget equation for the kinetic energy of the disturbance flow field:

A1 $$\begin{aligned} \frac{\mathrm{d}E(t)}{\mathrm{d}t} = q_{\text {dis}}+q_{\text {Rey}}+ q_{\text {inf}}. \end{aligned}$$

Here

A2 $$\begin{aligned} E(t) = \int \limits _{V}\frac{1}{2}(u^{n}_{i})^{2}\mathrm{d}V \end{aligned}$$

is the kinetic energy of the disturbance flow, where the velocity field $u^{n}_{i}$ is taken where fluid component *n* occupies a region in the volume *V*. On the right-hand side,

A3 $$\begin{aligned} q_{\text {dis}} = \frac{2}{\text {Re}}\int \limits _{V}-m_{n}e^{n}_{ij} e^{n}_{ij} \mathrm{d}V \end{aligned}$$

with the strain-rate tensor

A4 $$\begin{aligned} e^{n}_{ij}=\frac{1}{2}\left( \frac{\partial u^{n}_{i}}{\partial x_{j}}+\frac{\partial u^{n}_{j}}{\partial x_{i}}\right) , \end{aligned}$$

and

A5 $$\begin{aligned} q_{\text {Rey}} = \int \limits _{V}-{u}^{n}_{i}{u}^{n}_{j}\frac{\partial U^{n}_{i}}{\partial x_{j}}\mathrm{d}V \end{aligned}$$

with $U^{n}_{i}$ the velocity components of the base flow. When applying Gauss theorem, we also arrive at a term from the fluid interfaces,

A6 $$\begin{aligned} q_{\text {inf}} = \sum _{\forall S}\int \limits _{S}\sum _{S_{\pm }}{u}^{n}_{i}T^{n}_{ij}{N}^{n}_{j}\mathrm{d}S , \end{aligned}$$

with the stress tensor

A7 $$\begin{aligned} T^{n}_{ij}=-p^{n}\delta _{ij}+\frac{2m_{n}}{\text {Re}}e^{n}_{ij} , \end{aligned}$$

the components $N^{n}_{j}$ of the outward unit normal at the surface of fluid component *n*. The symbols $\sum _{\forall S}$ and $\sum _{S_{\pm }}$, respectively, indicate the summation over all interfaces and the two surfaces of the two fluid components separated by the interface. The surface term can be further evaluated by employing conditions for the stress tensor at the interface, where we index the two fluids by n=1,2. While the tangential stress is continuous at the interface,

A8 $$\begin{aligned} (\delta _{ij}-N_{i}N_{j})T^{1}_{jk}N_{k}=(\delta _{ij}-N_{i}N_{j})T^{2}_{jk}N_{k} , \end{aligned}$$

the normal stress difference is balance by the Laplace pressure,

A9 $$\begin{aligned} N_{i}T^{1}_{ij}N_{j}-N_{i}T^{2}_{ij}N_{j}=\Gamma \kappa , \end{aligned}$$

Here, $\Gamma$ is the rescaled surface tension as introduced in the main text and $\kappa$ the mean curvature.

Now, we can calculate $q_\text {inf}$ for the interfaces of the three-layer flow assuming only weakly perturbed interfaces so that the interface normal approximately points along the *y*-axis. Employing conditions (A8) and (A9) for the tangential and normal stresses, one obtains the final form $q_\text {inf} = q_{\text {nor}}+q_{\text {tan}}$ with

A10 $$\begin{aligned} q_\text {nor} = \sum _{\begin{array}{c} \text {both} \\ \text {interfaces} \end{array} } \int \limits _\text {S} \frac{\Gamma }{\text {Re}} \kappa u_{2} \mathrm{d}S \end{aligned}$$

and

A11 $$\begin{aligned} q_\text {tan} = \int \limits _{\text {S}-} (u_{1}^1-u_1^2)\tau _{xy}\mathrm{d}S + \int \limits _{\text {S}+} (u_{1}^2-u_1^1)\tau _{xy}\mathrm{d}S , \end{aligned}$$

where $\tau _{xy} = \tau ^{1}_{xy}=\tau ^{2}_{xy}$ with $\tau ^{n}_{xy}=\frac{m_{n}}{\text {Re}}\left( \frac{\partial u^{n}_{1}}{\partial y}+\frac{\partial u^{n}_{2}}{\partial x}\right)$ and $S\pm$ indicate the interfaces at $y=\pm t_s/2$. We also used the continuity of the *y* component of the velocity, $u_{2} = u_{2}^1 = u_{2}^2$, while the *x* components are different due to the viscosity contrast.
